# Liquid Crystal‐Driven Chemical Feeding Accelerates Condensation Reactions in Droplet Microreactors

**DOI:** 10.1002/advs.202504133

**Published:** 2025-09-14

**Authors:** Yang Xu, Alan H. Weible, Meng Zhang, Weichen Deng, Jen‐Chun Fang, Xiaoguang Wang

**Affiliations:** ^1^ William G. Lowrie Department of Chemical and Biomolecular Engineering The Ohio State University Columbus OH 43210 USA; ^2^ Sustainability Institute The Ohio State University Columbus OH 43210 USA

**Keywords:** chemical release, liquid crystals, lubricated surfaces, mass transport, microreactors

## Abstract

Droplet microreactors have gained significant attention as confined spaces for chemical reactions, offering enhanced reaction rates and control. However, the impact of mass transfer induced by chemical feeding within these microreactors remains largely unexplored. In this study, a novel approach is demonstrated using liquid crystal (LC) phase transitions to feed chemicals from a bulk LC film to droplet microreactors situated on the LC film. By manipulating LC mesophases, precise control is achieved over the solubility of chemicals within the bulk LC, enabling both in situ loading and controlled release. The results reveal that while droplet confinement inherently enhances reaction rates, the chemical feeding further improves mass transport within the droplet microreactors. This synergistic effect leads to a remarkable acceleration of chemical reactions, with conversion rates up to nine times higher than conventional bulk reactions. Furthermore, the broad applicability of this approach is demonstrated across various aldehyde reactions, the reusability of the LC films for multiple reaction cycles, and multi‐step microscale synthesis within a single droplet microreactor. These findings establish the LC system as an innovative platform for chemical feeding, substantially expanding the performance and utility of droplet microreactors for diverse chemical applications.

## Graphical abstract

This work introduces a liquid crystal (LC)‐based platform for dynamic chemical feeding in droplet microreactors, enabling unprecedented control over reaction kinetics and mass transfer. By leveraging LC phase transition‐mediated release, new pathways are unlocked for enhancing confined reaction environments, advancing the fundamental capabilities of microreactor technology.

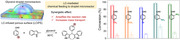

## 1 Introduction

Microdroplet reactors have attracted significant attention due to their ability to dramatically enhance reaction kinetics compared to bulk‐phase reactions, a concept first introduced by Cooks et al. in 2011.^[^ ^1^ ^]^ This enhancement stems from the intrinsic properties of microdroplets, particularly their high surface area‐to‐volume ratio.^[^ ^2^ ^]^ The increased surface area facilitates efficient mass and heat transfer, enabling faster reaction rates.^[^ ^3^ ^]^ Unique interfacial effects in microdroplets create reactive zones that concentrate specific ions or molecules, fostering conditions that are favorable for certain chemical transformations.^[^ ^4^ ^]^ These phenomena often lead to reaction pathways inaccessible in bulk solutions, resulting in increased reaction rates and selective reactions unachievable in traditional reactors.^[^ ^5^ ^]^ Pioneering work by Mazumdar and Zare et al. has demonstrated the profound impact of microdroplet confinement on reaction rates.^[^ ^6^ ^]^ For instance, the Pomeranz–Fritsch synthesis of isoquinoline in microdroplet reactors shows a reaction rate three orders of magnitude higher than in bulk solution, primarily due to the accumulation of surface charges.^[^ ^6a^ ^]^ Similar accelerations have been observed in other reactions, such as the Hantzsch reaction and base‐catalyzed Claisen–Schmidt condensation.^[^ ^6^, ^7^ ^]^ Despite these advantages, current microdroplet reactor systems typically introduce reactants prior to droplet formation. The possibility of adding reactants during the reaction process and its influence on mass transfer and reaction kinetics within microdroplets remains largely unexplored. This study seeks to address the unexplored potential of adding reactants during the reaction process within microdroplet systems, aiming to uncover how this dynamic approach can enhance mass transfer, accelerate reaction kinetics, and enable greater control over reaction outcomes.

Liquid crystals (LCs) are a class of anisotropic materials that exhibit properties between those of conventional liquids and solid crystals.^[^ ^8^ ^]^ Recent studies have shown that LCs can release the encapsulated chemicals on demand, a characteristic behavior observed in various configurations such as LC films submerged in bulk aqueous phases or water droplets resting on LC films.^[^ ^9^ ^]^ The key to this phenomenon lies in the unique phase transitions of LCs, which can be triggered by external stimuli such as temperature changes, electric fields, or light exposure.^[^ ^9a^ ^]^ When these transitions occur, they induce structural reorganizations within the LC matrix, facilitating the release of encapsulated molecules into the surrounding environment. This controlled release mechanism presents an exciting opportunity to achieve precise chemical feeding to droplet microreactors and study its effects on chemical reactions at the microscale. We hypothesize that LCs, serving as stimuli‐responsive substrates for droplet microreactors, could open new avenues for investigating mass transport phenomena and their impact on reaction kinetics. By leveraging the controlled release capabilities of LCs, we predict that the gradual introduction of reactants will significantly influence reaction rates and pathways within confined droplet environments. This could potentially enable the development of novel synthetic strategies and the design of advanced reactor platforms with controlled release properties.

In this work, we investigate the intricate relationship between chemical feeding, mass transport, and reaction kinetics within droplet microreactors situated on a novel LC film system containing pre‐encapsulated reactants. Our experimental model focuses on the in situ loading of 2‐methylindole within a LC film through phase transition, followed by its continuous feeding into a glycerol droplet microreactor containing 4‐nitrobenzaldehyde. The feeding process is achieved via thermal‐induced LC phase transitions, allowing for precise control over the release rate of the encapsulated reactant. Our findings reveal a synergistic effect between droplet confinement and reactant feeding. While the droplet environment itself contributes to increased reaction rates due to enhanced surface‐to‐volume ratios and unique interfacial phenomena, the continuous feeding of reactants from the LC substrate further accelerates the chemical reactions. This acceleration is primarily attributed to the significant enhancement of mass transfer within the glycerol droplets, facilitated by the constant influx of fresh reactants from the LC film. Importantly, we demonstrate that this chemical feeding‐induced increase in reaction rate is generalizable across a variety of aldehydes with different steric and electronic properties. Moreover, LC films with spatially patterned chemical loading allow programmable, on‐demand chemical release and multi‐step microscale synthesis, which are fundamentally inaccessible to conventional pre‐encapsulation strategies. These findings not only advance our understanding of microscale reaction dynamics but also pave the way for innovative approaches in controlled synthesis, drug delivery, and the development of smart materials with programmable release properties.

## 2 Results and Discussion

**Figure 1:**
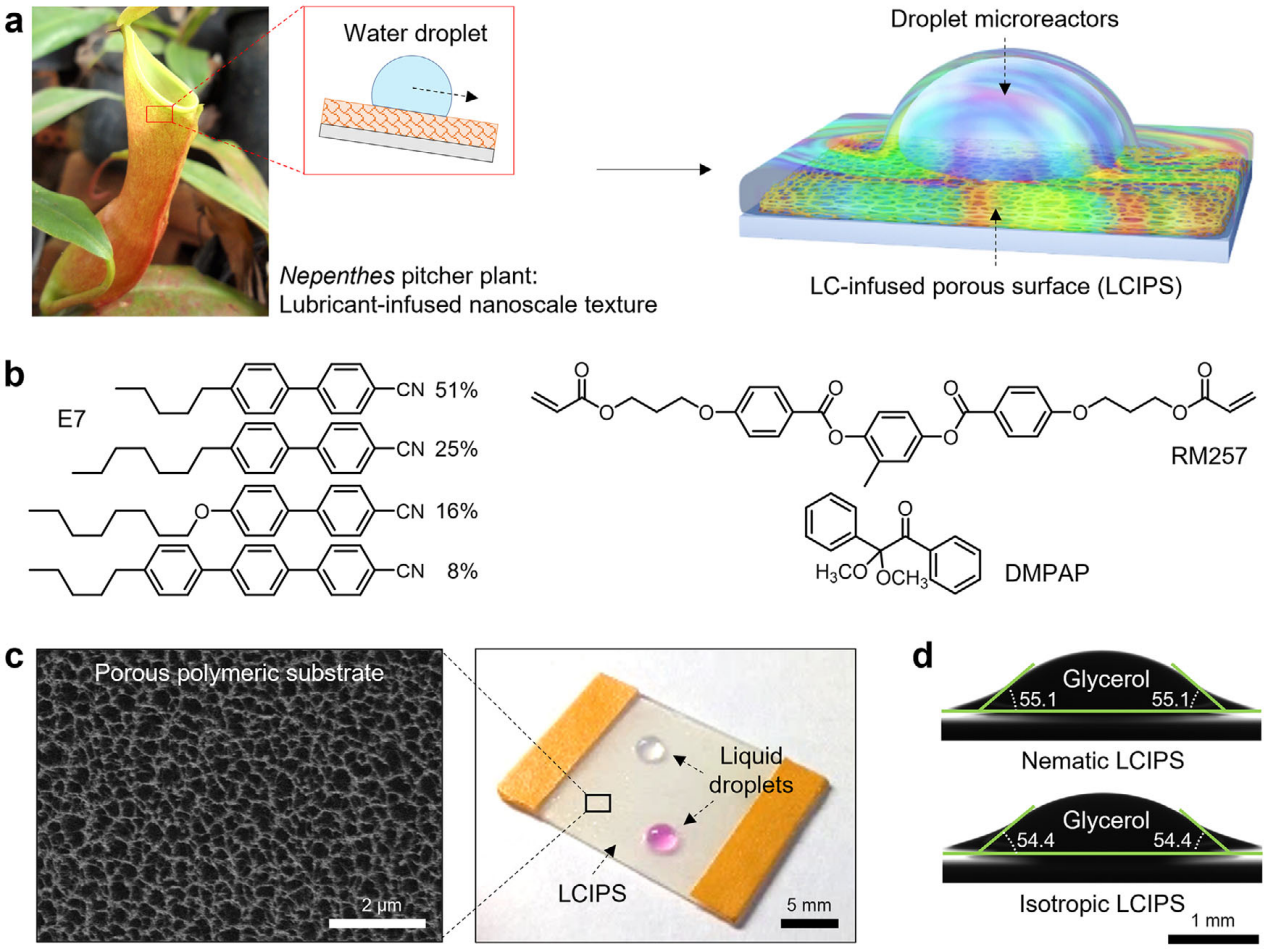
Design of LCIPS for droplet microreactors. a) Schematic illustration of a LCIPS inspired by the lubricant‐infused nanoscale texture of *Nepenthes* pitcher plant. Photo credit: The *Nepenthes* pitcher plant image is royalty‐free image from Pixabay company. b) Molecular structures of LC E7, crosslinker RM257, and photoinitiator DMPAP. c) Photograph of a LCIPS as the support for liquid droplets and a representative SEM image of the porous polymeric substrate without the infused LC. d) Contact angle goniometer images showing contact angles of glycerol droplet on LCIPS in nematic and isotropic phases. The micrographs were captured at 25 and 65 °C, corresponding to nematic and isotropic phases, respectively. The volumes of all droplets on LCIPS were 3 µL.

We developed a novel LC‐based reaction platform, LC‐infused porous surface (LCIPS), to support droplet microreactors by coating LC on a nanoporous polymer‐coated substrate (**Figure** **1**a). This approach effectively stabilized the LC film against dewetting caused by the droplet microreactors (see details in the Table S1, Supporting Information). We selected E7, a mixture of cyanobiphenyl and cyanoterphenol components, as the LC material due to its wide nematic phase temperature range (−62–62 °C), which allows for a broad spectrum of reaction temperatures (Figure 1b). In the nematic phase, E7's constituent molecules exhibit random spatial distribution with long‐range orientational order. Characterization of the substrate revealed that the porous poly(1,4‐bis[4‐(3‐acryloyloxypropyloxy)benzoyloxy]‐2‐methylbenzene) (polyRM257) network had pore sizes ranging from 200 to 500 nm, as measured by scanning electron microscopy (SEM; Figure 1c). The E7 film coated on this network achieved a thickness of 160 µm. We selected glycerol as the droplet microreactor medium due to its high solubility and low volatility, properties that make it ideal for various chemical reactions. Contact angle measurements showed that glycerol microdroplets on the E7 film exhibited apparent contact angles of 55.1° and 54.4° in the nematic and isotropic phases, respectively (Figure 1d; Figure S1, Supporting Information).

Previous research has demonstrated that micrometer‐sized liquid droplets dispersed in nematic phase LCs induce reorientation of the surrounding LC molecules.^[^ ^9a^ ^]^ This reorientation leads to the formation of topological defects, which prevent droplet coalescence and their escape from the bulk LC to the environment. Interestingly, when the LC undergoes a phase transition from nematic to isotropic, the ordered structure is disrupted, allowing the encapsulated microdroplets to be released from the bulk LC into the aqueous phase above (either bulk water or water droplets). This controlled release mechanism offers a promising approach for chemical feeding to droplet microreactors on a LCIPS.^[^ ^9c,e^ ^]^

It is worth noting that past studies typically dispersed microdroplets within a bulk LC phase before coating this mixture onto a porous polymer‐coated substrate.^[^ ^9c^ ^]^ However, this method necessitates repeated rinsing and coating of LC films, limiting its practicality and reusability. Our approach aims to achieve in situ loading and release of chemicals across the LCIPS, enabling cyclic utilization and improving overall efficiency. Previous investigations have shown that glycerol is partially miscible with isotropic phase LCs, such as E7 and 4‐cyano‐4′‐pentylbiphenyl (5CB).^[^ ^10^ ^]^ Importantly, the solubility of glycerol in the nematic phase LC significantly decreases, resulting in phase separation between glycerol and the nematic‐phase LC (Figure S2, Supporting Information). This phenomenon leads to the formation of micrometer‐sized glycerol droplets within the bulk LCIPS. Building on these findings, we sought to use glycerol to achieve in situ loading and release of chemicals within the LCIPS, potentially offering a more versatile and efficient approach to droplet microreactor systems.

**Figure 2:**
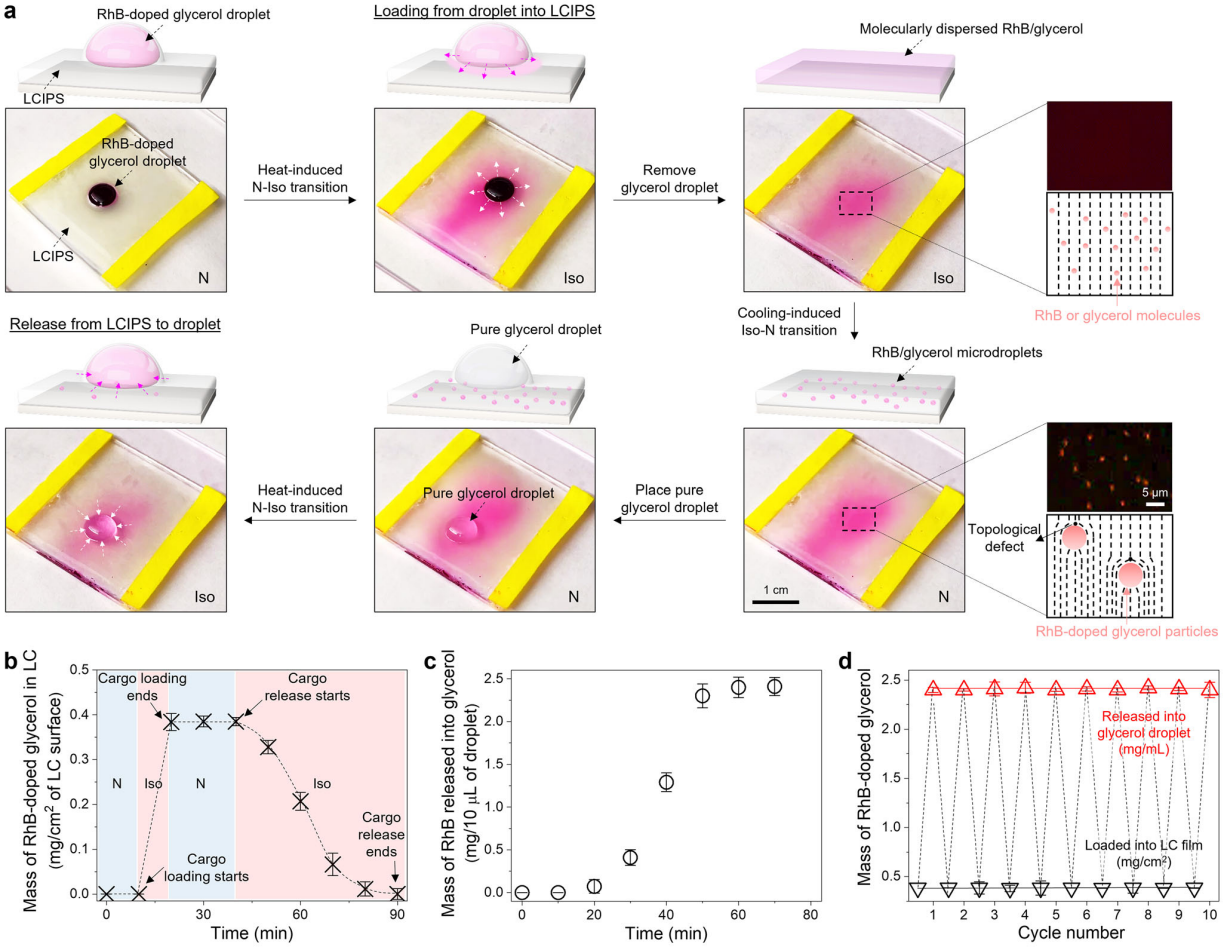
In situ loading and release of chemicals on LCIPS. a) Sequential photographs and corresponding schematics illustrating in situ chemical loading and release between glycerol droplet and LCIPS. The concentration of RhB in the glycerol droplet was 100 mg mL^−1^. The duration of the chemical‐loaded and released LCIPS stayed in the isotropic phase, was 10 and 50 min, respectively. Insets show the schematic illustrations and corresponding fluorescence micrograph of the size‐dependent LC ordering around the chemicals in an optical cell prepared by pairing two polyimide‐coated glass slides (see details in the Supporting Information). The black dashed lines indicate the local average direction of the long molecular axes of the LCs (namely, the director). b) Plot showing the mass of RhB‐doped glycerol in LC as a function of the time of chemical loading and release between droplet and LCIPS. c) Plot showing the mass of RhB released into glycerol on LCIPS as a function of the time LCIPS was in the isotropic phase. d) Cycling tests of chemical loading and release between droplet and LCIPS. The temperature was set to 25 and 65 °C to achieve nematic and isotropic phase of E7, respectively. Error bars represent standard deviations and *n* = 3 for each data point.

**Figure** **2**a illustrates the dissolution of a 10‐µL Rhodamine B (RhB)‐doped glycerol droplet into an isotropic E7 film when heated to 65 °C for 5 min. Fluorescence microscopy revealed a uniform red coloration in the E7 film beneath the glycerol droplet, indicating effective molecular dispersion of RhB and glycerol (Figure S3, Supporting Information). Upon cooling the LCIPS back to the nematic phase, we observed the formation of distinct red droplets with an average diameter of 2.0 ± 0.5 µm, suggesting phase separation of RhB‐doped glycerol from the nematic E7. We measured the loading capacity of RhB‐doped glycerol in a 1 cm^2^ area of the LCIPS to be 0.38 mg (Figure 2b), demonstrating that LC mesophase‐dependent glycerol's solubility allows for efficient in situ chemical loading.

Next, we tested the release of these loaded glycerol microdroplets. A 10‐µL droplet of pure glycerol was placed on a nematic E7 film containing RhB‐doped glycerol microdroplets. Initially, no measurable release occurred for at least 20 min, indicating colloidal stability of glycerol microdroplets in the nematic phase. However, upon heating the E7 film to induce a transition to the isotropic phase, we observed continuous release of RhB‐doped glycerol into a millimeter‐sized glycerol droplet on top of the E7 film. Approximately 2.48 mg of encapsulated RhB‐doped glycerol was released within 60 min (Figure 2c). The amount released could be controlled by adjusting the time spent in the isotropic phase. To assess the reusability of our system, we performed multiple cycles of in situ loading and release. Our results demonstrate that this process can be repeated for at least 10 cycles without significant loss of efficiency (Figure 2d), indicating the robustness and potential long‐term applicability of our approach. We note here that due to the slipperiness of the nematic and isotropic‐phase LC, glycerol droplets on the LCIPS can be freely moved by simply tilting the film.

To understand the phase‐dependent chemical release behavior in the LCIPS systems, we developed a modified Dejaguin–Landau–Verway–Overbeek model through the combination of van der Waals force (*F*_vdW_), capillary force (*F*_cap_), electric double layer force (*F*_edl_), and LC orientational order‐originated elastic force (*F*_el_) (see details in Supporting Information). The net force (*F*_net_) acting on the glycerol microdroplet is written as:

(1) $$\begin{array}{ccc} F_{net} & = & F_{vdW}+F_{cap}+F_{edl}+F_{el}=-\frac{A_{\mathrm{Gly}-\mathrm{LC}-\mathrm{Gly}}R_{\mathrm{Gly}\;\mathrm{mdrop}}}{6x^{2}}\\ & & -\frac{2\gamma_{\mathrm{LC}}\xi \pi R_{\mathrm{Gly}\;\mathrm{mdrop}}^{3}}{{\left(R_{\mathrm{Gly}\;\mathrm{reactor}}+x\right)}^{2}}+64\pi \varepsilon_{\mathrm{LC}}\varepsilon_{0}{\left(\frac{k_{B}T}{e}\right)}^{2}\tanh\\ & & \left(\frac{\mathrm{ez}\psi_{\mathrm{Gly}\;\mathrm{reactor}}}{4k_{B}T}\right)\tanh\left(\frac{\mathrm{ez}\psi_{\mathrm{Gly}\;\mathrm{mdrop}}}{4k_{B}T}\right)R_{\mathrm{Gly}\;\mathrm{mdrop}}\exp\left(-\kappa x\right)\\ & & +\frac{\pi \alpha^{2}\beta {\mathrm{KR}}_{\mathrm{Gly}\;\mathrm{mdrop}}^{4}}{{\left(R_{\mathrm{Gly}\;\mathrm{mdrop}}+x\right)}^{4}} \end{array}$$

where *A*_Gly–LC–Gly_ is the Hamaker constant for the interaction between two glycerol phases across the LC, *R*_Gly mdrop_ is the radius of the encapsulated glycerol microdroplet, *x* is the surface‐to‐surface distance between the encapsulated glycerol microdroplet and glycerol droplet microreactor, *γ*_LC_ is the surface tension of the LC, *ξ* is a coefficient to estimate the average hydrostatic pressure acting on teach hemisphere, *ε*_0_ is the vacuum permittivity, *ε*_LC_ is the relative permittivity of the LC, *k*_B_ is the Boltzmann constant, *T* is the temperature, *z* is the valence number of the dominant ionic species, *e* is the elementary charge, *κ* ^−1^ is the Debye length, *ψ*_Gly mdrop_ is the zeta potential of the encapsulated glycerol microdroplet, and *ψ*_Gly reactor_ is the zeta potential of the glycerol droplet microreactor. *α* and *β* are material constants of the LC, and *K* is the Frank elastic constant of the LC. Our thermodynamic model shows that the nematic‐to‐isotropic transition eliminates *F*_el_, resulting in an attractive *F*_net_ that drives glycerol microdroplet release (Figure S4, Supporting Information), which supports our experimental observation of phase transition‐triggered chemical release on the LCIPS. In addition, the threshold temperature required to activate the release of encapsulated glycerol microdroplets can be adjusted by tuning the nematic–isotropic phase transition temperature (*T*_N–I_) of the LC. For example, we observed chemical release from 5CB films at 38 °C (*T*_N–I_,_5CB_ is ≈35 °C; Figure S5, Supporting Information).

The above observations suggest a promising avenue for achieving in situ chemical loading and feeding via manipulation of LC mesophases. To study how the chemical feeding process influences reaction kinetics within droplet microreactors, we examined the kinetics of chemical reactions in glycerol droplet microreactors on LCIPS. It is widely recognized that glycerol is an effective green solvent for bulk liquid‐phase catalytic and non‐catalytic organic synthesis due to its high boiling point (290 °C), making the development of organic reactions at high temperatures technically more feasible, even in open surface systems.^[^ ^11^ ^]^ However, to date, the chemical reactions in glycerol droplet microreactors on open surfaces have not been reported.

**Figure 3:**
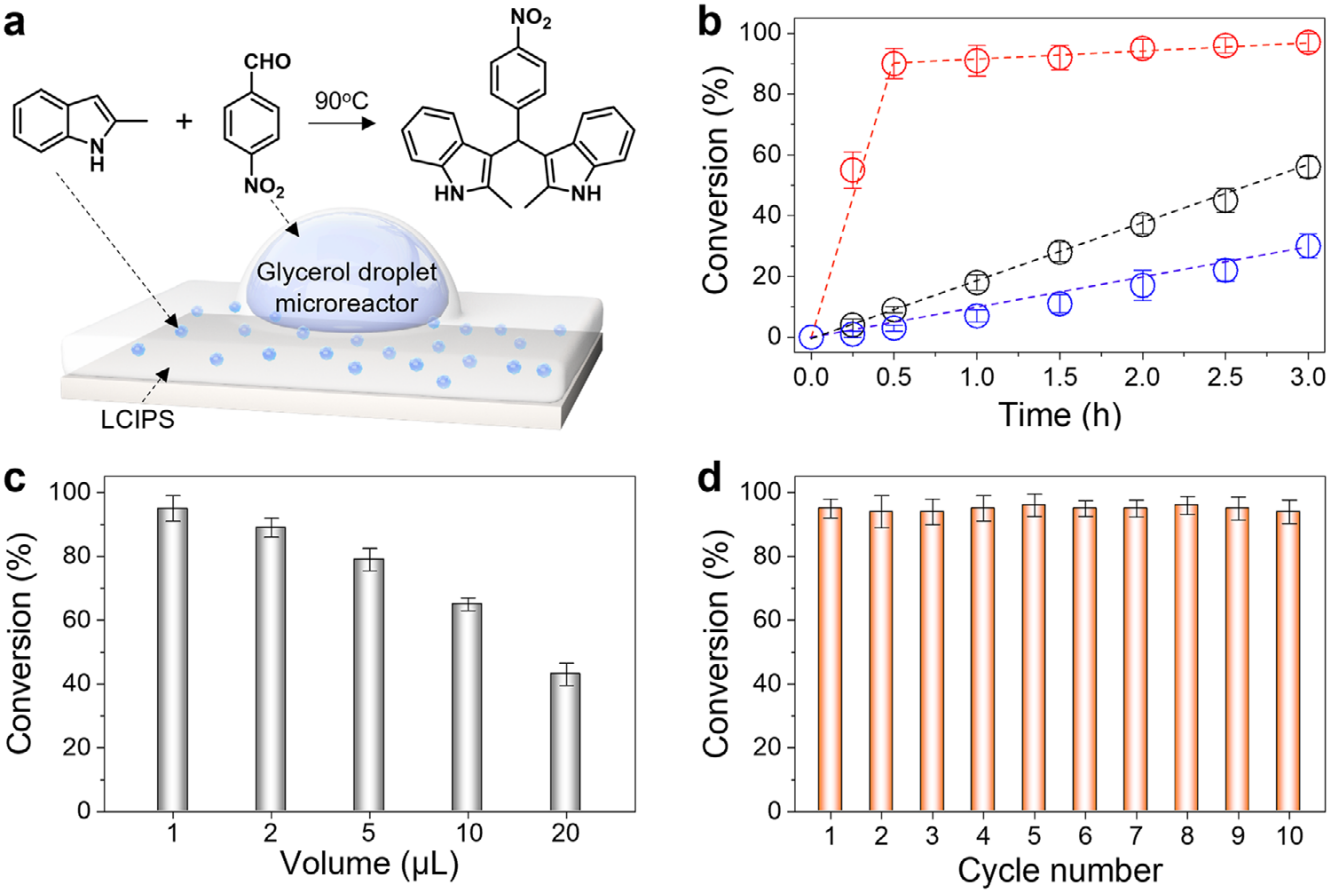
LCIPS‐mediated reactant release for condensation reactions in droplet microreactors. a) Schematic of the condensation reaction between 4‐nitrobenzaldehyde and 2‐methylindole in glycerol droplet microreactors on LCIPS. b) Conversion of 4‐nitrobenzalde over time for different droplet microreactors. Red: 1‐µL glycerol droplet containing 4‐nitrobenzaldehyde on LCIPS loaded with 2‐methylindole; black: 1‐µL glycerol droplet containing both reactants on pure LCIPS; blue: 10‐µL glycerol droplet containing 4‐nitrobenzaldehyde on LCIPS loaded with 2‐methylindole. c) Conversion of 4‐nitrobenzaldehyde as a function of microreactor volume. d) Cycling tests of the condensation reaction, showing the conversion of 4‐nitrobenzaldehyde in droplet microreactors on LCIPS over multiple cycles. Reactions were initiated by placing glycerol droplets on LCIPS and rapidly heating the system to 90 °C. The initial amounts of 2‐methylindole and 4‐nitrobenzaldehyde in the LCIPS or glycerol droplet reactor were 2.5 mmol cm^−2^ and 1 mmol, respectively. Reactant 2‐methylindole was reloaded between each cycle. Error bars represent standard deviations, with *n* = 3 for each data point.

To test our hypothesis, we conducted a detailed investigation of a condensation reaction between 4‐nitrobenzaldehyde and 2‐methylindole using glycerol droplet microreactors on E7 film (**Figure** **3**a). Our experimental design involved a precise and controlled approach to chemical loading and reaction monitoring. We first in situ loaded 2.5 mmol cm^−2^ of 2‐methylindole within a nematic‐phase LCIPS. A 1‐µL glycerol microdroplet containing 1 mmol of 4‐nitrobenzaldehyde was then placed on the LCIPS. To initiate the reaction and trigger the release of 2‐methylindole, we gradually increased the temperature to 90 °C, which induced a nematic‐to‐isotropic phase transition (see Figure S6, Supporting Information for the mass released as a function of time in the isotropic phase). To comprehensively understand the role of chemical feeding in our system, we designed two additional sets of control experiments. The first control experiment involved placing a 1‐µL glycerol microdroplet containing both reactants (1 mmol 4‐nitrobenzaldehyde and 2.5 mmol 2‐methylindole) directly on the pure LCIPS without stirring. In the second control, a larger 10‐µL glycerol microdroplet was placed on a nematic LC film pre‐loaded with 2.5 mmol cm^−2^ of 2‐methylindole. Both control experiments were conducted at a consistent temperature of 90 °C to ensure comparable reaction conditions.

The experimental results, illustrated in Figure 3b,c, revealed four significant and interconnected observations that provide insights into the reaction dynamics within droplet microreactors on the LCIPS platform. First, after 30 min of reaction at 90 °C, the reaction conversion of 4‐nitrobenzaldehyde and 2‐methylindole in the 1‐µL glycerol droplet microreactors without chemical feeding reached 10%. Notably, this conversion was almost twice that observed in the 10 µL glycerol droplet on the LCIPS. The enhanced reaction rate observed within glycerol droplet microreactors can be attributed to the strong affinity of indole derivatives for glycerol,^[^ ^11b^ ^]^ as well as chemical enrichment at the glycerol–LC interface or confinement‐induced reactivity. This finding suggests that microdroplet confinement itself can efficiently accelerate condensation chemical reactions, a phenomenon consistent with previous observations in droplet microreactor systems.^[^ ^1^, ^2^ ^]^ In addition, the cycling tests of the condensation reaction show the conversion of 4‐nitrobenzaldehyde in droplet microreactors on LCIPS over multiple cycles, demonstrating the reusability of LCIPS platform for controlled chemical release to droplet microreactors (Figure 3d).

The second and the most striking observation emerged from the chemical feeding experiment. After 30 min at 90 °C, the reaction conversion in the 1‐µL glycerol‐based droplet microreactors with LCIPS‐mediated chemical feeding reached 90%, a ninefold increase compared to the droplet microreactors without chemical feeding. This substantial enhancement demonstrates that chemical feeding process can dramatically accelerate chemical reactions within confined droplet environments. In addition, the outcomes of this reaction were independent of the sequence of adding reagents (Figure S7, Supporting Information). We hypothesize that this acceleration is attributed to enhanced mass transfer within the droplet microreactors, driven by continuous release of reactants from the LCIPS.

**Figure 4:**
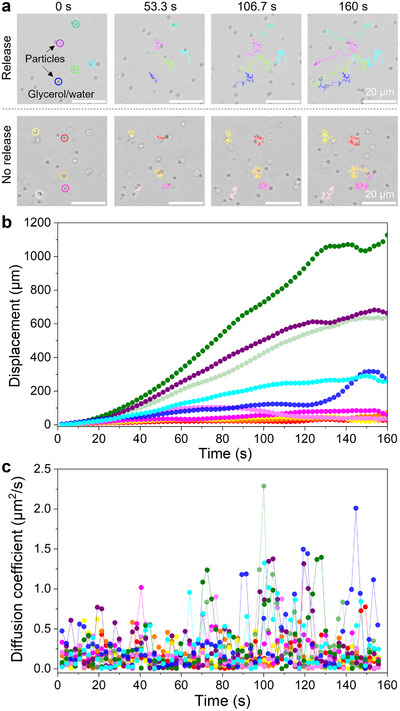
Chemical release‐enhanced mass transfer. a) Micrographs showing 1‐µm polystyrene particle trajectories in a glycerol/water (50/50 vol%) film in contact with an LC film at 70 °C (see Movie S1, Supporting Information). b) Particle displacement over time. c) Instantaneous diffusion coefficient of particles as a function of time. Red, orange, yellow, pink, and light pink curves represent the pure LC film (control system), while mint, purple, green, blue, and dark blue curves correspond to the LC film with encapsulated glycerol microdroplets. Upon heating to 70 °C, the nematic–isotropic phase transition triggers the release of glycerol microdroplets, inducing convective flow and enhancing mass transfer. The volume fraction of glycerol microdroplets in E7 was 10 vol%, with 10 mmol SDS added to the glycerol microdroplets to enhance colloidal stability and prevent coalescence within bulk E7.

To test this hypothesis, we examined the effect of chemical release on mass transfer in bulk glycerol by tracking the motion of tracer particles in a glycerol film in contact with an LC film (**Figure** **4**a,b). When the LC film contained encapsulated glycerol microdroplets, heating above the nematic–isotropic phase transition temperature (70 °C) triggered their release. This release induced convective flow, resulting in significant instantaneous particle displacement within the glycerol film. In contrast, when the LC film lacked encapsulated glycerol and was subjected to the same heating protocol, no chemical release occurred, and particles exhibited only Brownian motion.^[^ ^12^ ^]^ To quantify this effect, we estimated the time‐dependent diffusion coefficients of the particles in both conditions (Figure 4c; see details in the Supporting Information). In the control system, the diffusion coefficient remained consistent with Brownian motion. However, in the chemical release system, convective flow from the sudden glycerol release led to a pronounced increase in diffusion coefficient. These findings validate our hypothesis and provide a mechanistic explanation for the observed enhancement in reaction rates within droplet microreactors.

**Figure 5:**
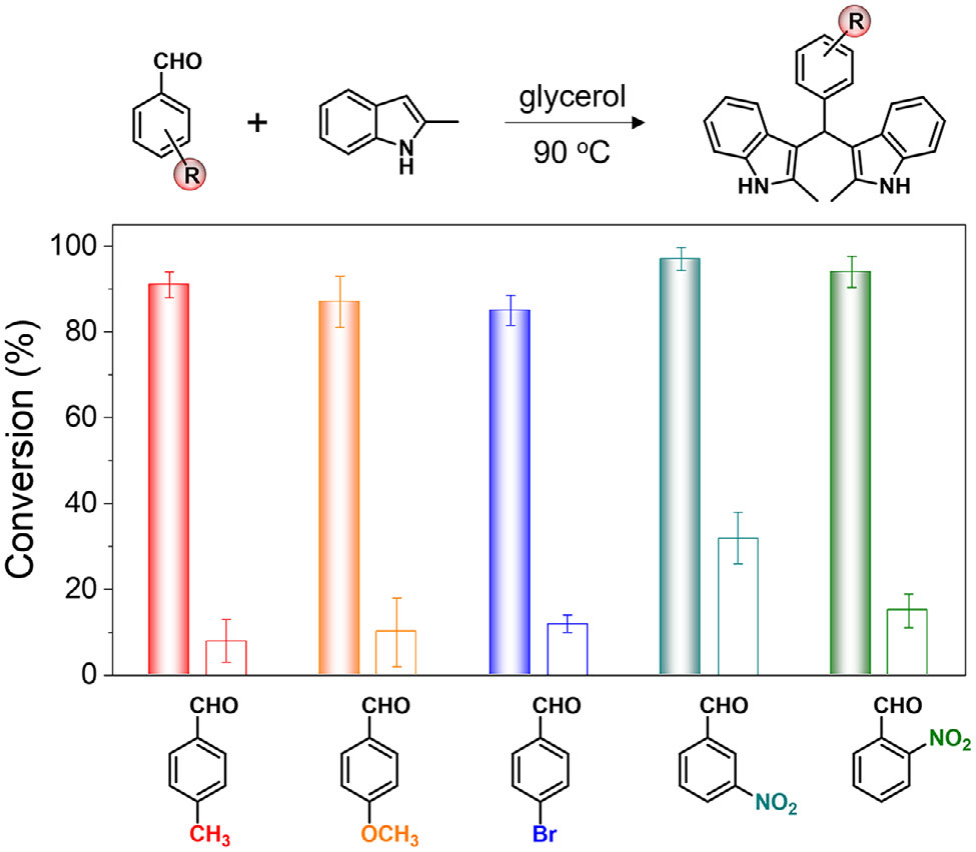
Generalizability of the chemical feeding‐induced acceleration within droplet microreactors on LCIPS. Plot showing the conversion of a variety of aldehydes in glycerol droplet microreactors with (solid columns) or without (hollow columns) chemical feeding. In a typical chemical feeding experiment, a 1‐µL glycerol microdroplet containing 1 mmol of aldehydes was placed on LCIPS preloaded with 2.5 mmol cm^−2^ of 2‐methylindole. In contrast, control experiments without chemical feeding involved a 1‐µL glycerol microdroplet containing both aldehyde and 2‐methylindole. The reactions were conducted at a consistent temperature of 90 °C for 30 min to ensure comparable reaction conditions. Error bars represent standard deviations and *n* = 3 for each data point.

Our third key finding addresses the generalizability of the chemical feeding‐induced acceleration across different chemical systems. As shown in **Figure** **5**, we tested the reaction with a variety of aldehydes in glycerol droplet microreactors. Remarkably, all tested chemical reactions achieved conversion rates exceeding 85% after 30 min (see detailed nuclear magnetic resonance (NMR) analysis in the Supporting Information). These conversion rates were consistently 3 to 10 times higher than those observed in droplet microreactors without chemical feeding.

Fourth, our findings highlight the exceptional durability of the LCIPS platform. The LCIPS demonstrated reusability for at least 10 consecutive reaction cycles without any noticeable decline in catalytic efficiency. Moreover, a TiO_2_‐mediated photocatalytic degradation of RhB‐doped glycerol microdroplets on the LCIPS also demonstrated the cleaning ability of the LCIPS platform (Figure S8, Supporting Information). In addition, we note that our LC‐mediated chemical feeding strategy is effective for diffusion‐limited reactions, where reaction rates are primarily governed by mass transport rather than activation energy barriers (Figure S9, Supporting Information). This robustness demonstrates the potential of LCIPS to enable sustainable and reliable catalytic micro‐reactions, with promising implications for both research advancements and practical applications.

**Figure 6:**
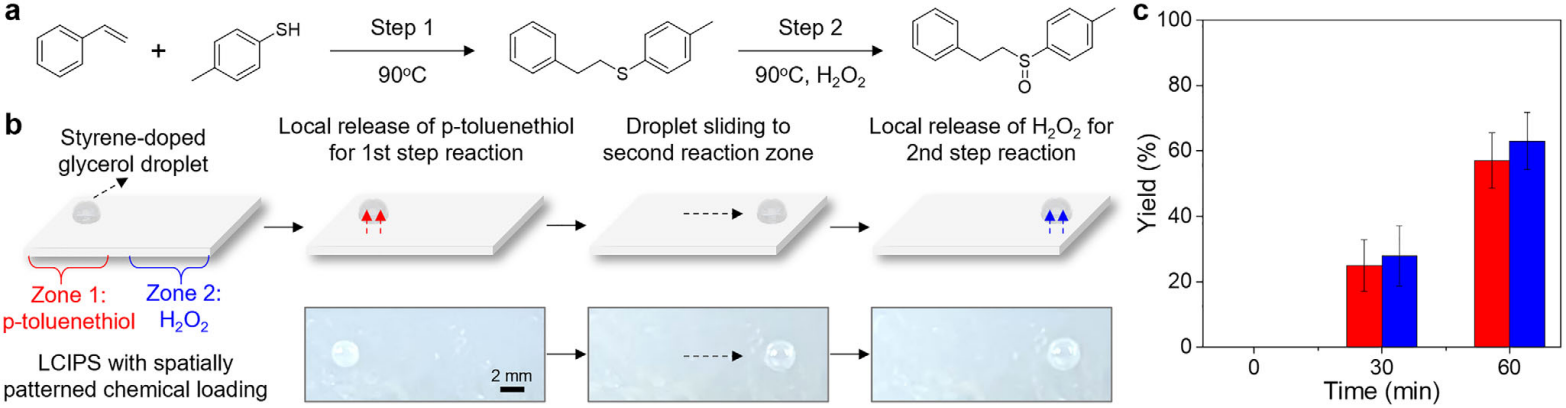
Two‐step cascade reaction within a single glycerol droplet microreactor achieved by spatially patterned chemical loading on LCIPS. a) Chemical reaction scheme. Step 1: thiol–ene coupling of styrene (preloaded in droplet) with *p*‐toluenethiol (released from LCIPS Zone 1) to yield phenethyl(*p*‐tolyl)sulfane; Step 2: oxidation of phenethyl(*p*‐tolyl)sulfane with H_2_O_2_ (released from LCIPS Zone 2) to produce 1‐methyl‐4‐(phenethylsulfinyl)benzene. b) Schematic illustration and photographs showing the droplet at Zone 1 (Step 1), droplet sliding to Zone 2, and subsequent Step 2 reaction. c) Reaction yields for both steps after 30 min at 90 °C (red: phenethyl(*p*‐tolyl)sulfane; blue: 1‐methyl‐4‐(phenethylsulfinyl)benzene). Error bars represent standard deviations, and *n* = 3 for each data point.

To further demonstrate the unique capability of LCIPS for programmable, multi‐step microscale synthesis, we designed a two‐step cascade reaction within a single glycerol droplet microreactor by employing spatially patterned chemical loading on the LCIPS (**Figure** **6**a). As schemed in Figure 6b, in the first step, the glycerol droplet, preloaded with styrene, was positioned on Zone 1 of the LCIPS, which locally released *p*‐toluenethiol upon heating above *T*_N–I_, yielding phenethyl(*p*‐tolyl)sulfane. The droplet was then slid to Zone 2 by tilting the substrate, where localized release of H_2_O_2_ triggered the second reaction, oxidizing phenethyl(*p*‐tolyl)sulfane to 1‐methyl‐4‐(phenethylsulfinyl)benzene. NMR spectroscopy confirmed both intermediates and the final product (see details in Supporting Information). The yields of each step demonstrate that LCIPS enables precise temporal and spatial control over sequential reagent delivery in a single droplet microreactor (Figure 6c). This capability is fundamentally inaccessible to conventional pre‐encapsulation strategies, where all reagents must be preloaded prior to initiating the reaction.^[^ ^1^, ^2^, ^3^, ^4^, ^5^, ^6^, ^7^ ^]^

## 3 Conclusion

In conclusion, our study presents an innovative approach to droplet microreactor technology by utilizing the unique properties of LCs for controlled chemical feeding. We have demonstrated that LC‐mediated chemical feeding not only enhances reaction rates through confinement effects but also significantly improves mass transfer within droplet microreactors. The versatility of our approach is evident in its applicability to a wide range of aldehyde reactions and the remarkable reusability of the LCIPS. These features position our system as a powerful tool for both fundamental research in reaction kinetics and practical applications in synthetic chemistry.

This work will open new avenues for research and development. The precise control over chemical feeding enabled by LC phase transitions can lead to the design of more complex, multi‐step reaction sequences within single droplet environments. Moreover, the principles established here could be extended to other classes of responsive materials, potentially revolutionizing fields such as drug delivery, materials synthesis, and environmental remediation. As we continue to explore the full potential of this LCIPS‐based microreactor system, we anticipate that it will not only advance our understanding of confined chemical reactions but also pave the way for new, more efficient processes in chemical synthesis and analysis. Future efforts will seek to use LC‐mediated chemical feeding to conduct different classes of chemical reactions in droplet microdroplets, including those known to be challenging in bulk systems, such as oximation reactions,^[^ ^13^ ^]^ redox reactions,^[^ ^14^ ^]^ and coupling reactions.^[^ ^15^ ^]^ This work represents a significant step toward more sustainable and controllable chemical technologies, aligning with the broader goals of green chemistry and process intensification.

## Conflict of Interest

The authors declare no conflict of interest.

## Supporting information



Supporting Information

Supplemental Movie 1

## Data Availability

The data that support the findings of this study are available from the corresponding author upon reasonable request.
