# Supplementary material for: Liquid Crystal‐Driven Chemical Feeding Accelerates Condensation Reactions in Droplet Microreactors
Source: Adv Sci (Weinh). 2025 Sep 14;12(45):e04133. doi: 10.1002/advs.202504133 (PMC12677612; doi:10.1002/advs.202504133)
Supplement: Supplementary file 1 — Supporting Information [file ADVS-12-e04133-s002.docx]

Supporting Information

Liquid Crystal-Driven Chemical Feeding Accelerates Condensation Reactions in Droplet Microreactors

Yang Xu,^1#^ Alan H. Weible,^1#^ Meng Zhang,^1^ Weichen Deng,^1^ Jen-Chun Fang,^1^ Xiaoguang Wang^1,2^*

**Materials and Methods**

**Materials**

The following LC chemicals were purchased from Synthon Chemicals GmbH & Co. KG: E7 and 1,4-bis-[4-(3-acryloyloxypropyloxy)benzoyloxy]-2-methylbenzene (RM257). The following chemicals were purchased from Sigma-Aldrich: Photoinitiator 2,2-dimethoxy-2-phenylacetophenone (DMPAP), Rhodamine B (RhB), glycerol, ethanol, dimethyloctadecyl[3-(trimethoxysilyl)propyl]ammonium chloride (DMOAP), 4-nitrobenzaldehyde, 2-nitrobenzaldehyde, 3-nitrobenzaldehyde, 4-methylbenzaldehyde, 4-methoxybenzaldehyde, 4-bromobenzaldehyde, 2-methylindole, and titanium dioxide (TiO_2_) nanopowder/nanoparticles. Plain microscope slides and 1 µm-in-diameter polystyrene microsphere suspension were purchased from Fisher Scientific. Unless stated otherwise, purchased chemicals and materials were used as received without further modification or purification.

**Preparation of DMOAP-functionalized glass slides**

First, glass slides were rinsed with water and ethanol and dried under a stream of nitrogen gas. Then, the cleaned glass slides were placed in a 1% v/v DMOAP silane water solution for 15 min. Afterwards, the glass slides were washed first with water and then with ethanol to remove unreacted DMOAP molecules. Subsequently, the DMOAP-functionalized glass slides were dried using nitrogen gas. These slides were stored in a dark room at ambient pressure and temperature to prevent light from damaging the DMOAP coating.

**Preparation of LC-infused porous surface (LCIPS)**

To make LCIPS against dewetting by glycerol, we prepared a LC mixture consisting of E7 (90 wt%) and RM257 (10 wt%). The photoinitiator DMPAP was added to the mixture at 1 wt% based on the total mass of the LC mixture. Next, the uniform mixture (150 μL) was spread evenly on a 2.5 cm × 2.5 cm DMOAP-functionalized glass slide. Subsequently, the LC mixture-covered glass slide was exposed to a UV lamp (Spectroline, EA-140; 365 nm) at 2.0 mW/cm^2^ for 30 min at 35^o^C. A semitransparent E7-infused polyRM257 porous surface was made with 160 μm thickness. Finally, we drop-cast the same non-reactive LC E7 (80 μL) onto the E7-infused polyRM257 nanoporous structure to form a 130 μm-thick E7 lubricating film.

***In-situ* loading and release of chemicals from the LCIPS**

We mixed 10 mg RhB dye and 1 mL glycerol at 2,000 rpm to prepare the RhB/glycerol mixture. For the loading process, we dropped a 10-μL RhB/glycerol mixture droplet on the LCIPS. Then, we increased the temperature from 25^o^C (nematic) to 65^o^C (isotropic) and kept the film in the isotropic phase for 10 min to allow RhB/glycerol mixture to diffuse in the E7 LCIPS. For the release process, after depositing a 10-μL droplet of pure glycerol, we increased the temperature from 25^o^C to 65^o^C and maintained the film in the isotropic phase for 50 min to facilitate the release of microdroplets of RhB/glycerol from the LCIPS to the glycerol droplet.

**Characterization of the morphology of porous polyRM257 substrates**

The porous polyRM257 substrate used for the scanning electron microscopy (SEM) imaging was prepared from the photopolymerization of a mixture of 10 wt% RM257 in E7 followed by an extraction of the nonreactive E7 with ethanol. After being fully dried, the porous substrate was coated with a thin layer of gold before imaging. Finally, the morphology of the porous polyRM257 substrate was imaged using an FEI Quanta 200 SEM with an acceleration voltage of 5 kV at a working distance of approximately 9 mm (Fig. 1c in the main text).

**Interfacial tension and contact angle measurement**

Contact angles were measured using the sessile drop method and interfacial tensions were measured using the pendant drop method. A KRÜSS DSA 100 goniometer was used for all measurements. The sessile drop method measurements were made using 2-µL glycerol droplets that were deposited on the LCIPS. For the pendant drop method measurements, different liquids were pushed at a rate of 5 μL/min through a needle to minimize the effect of dynamic forces on the shape of the droplet. The interfacial tensions were calculated from approximately 10 measurements taken from three different droplets. Glycerol was placed in a quartz cell with the LC being placed in a syringe with a needle held under the surface of the glycerol to determine the glycerol–E7 interfacial tension. The goniometer is equipped with a high-resolution camera that was used to capture images of the droplets that were then analyzed with built-in software to calculate the surface tensions. The data can be found in Table S1.

**Chemical loading and release determined by gravimetry**

Chemical loading and release on LCIPS were quantified using gravimetry. For chemical loading, we first measured the mass of the LCIPS on a DMOAP-functionalized glass substrate (m_1_ per 1 cm^2^ LC surface) using a Mettler Toledo analytical balance. A chemical-loaded glycerol droplet was then placed onto the LCIPS. After the active loading of glycerol and chemical into the LCIPS, the excess droplet was removed, and the mass of the LCIPS was measured again (m_2_ per 1 cm^2^). The mass of the loaded glycerol/chemical in the LCIPS was determined as *m*_2_ – *m*_1_, and the mass of the loaded chemical was determined as (*m*_2_ *– m*_1_) *x*, where *x* is the mass fraction of the chemical in glycerol.

For chemical release, a pure glycerol droplet was placed onto the chemical-loaded LCIPS. Upon activation, the chemical and glycerol microdroplets were released into the glycerol droplet. After removing the droplet, the mass of the LCIPS was measured again (m_3_). The released glycerol/chemical mass was calculated as *m*_2_ *– m*_3_, and the mass of the released chemical was determined as (*m*_2_ *– m*_3_) *x*.

**UV–visible spectrophotometry measurement**

The concentration of RhB dyes in glycerol reactor droplets was determined using a PerkinElmer Lambda 950 UV–visible spectrophotometer. Two 10-µL glycerol droplets, one containing 1 wt% TiO_2_ particles and the other without, were placed on nematic E7 films. The concentration of RhB dyes within the LCIPS was measured to be 1 mmol/cm^2^. To initiate the reaction and release the pre-loaded RhB dyes, the temperature was raised to 90°C, inducing a controlled nematic-to-isotropic phase transition. After TiO_2_-mediated photocatalytic degradation of RhB within glycerol droplets on the LCIPS, we withdrew 2 μL of aliquots from the droplet, placed them into UV cuvettes, and diluted them with 998 μL of water. Then, we measured the UV–visible absorption spectra ranging from 200 nm to 800 nm.

**Condensation reactions within glycerol droplet microreactors on LCIPS**

To demonstrate chemical reactions in droplet microreactors on LCIPS, we utilized a condensation reaction between aldehydes and 2-methylindole. Taking the reaction between 4-nitrobenzaldehyde and 2-methylindole as an example, a 10-µL glycerol droplet containing 10 mmol of 2-methylindole was placed onto the LCIPS. To facilitate the *in-situ* loading of 2-methylindole into the LCIPS, the system was gradually heated to 90°C, inducing a controlled nematic-to-isotropic phase transition. After 10 min of activated loading, the glycerol droplet was removed, and the concentration of 2-methylindole within the LCIPS was measured to be 2.5 mmol/cm^2^.

Subsequently, a 1-µL glycerol microdroplet containing 1 mmol of 4-nitrobenzaldehyde was placed onto the LCIPS. To initiate the reaction and release the pre-loaded 2-methylindole, the temperature was again raised to 90°C, inducing a second controlled nematic-to-isotropic phase transition. The reaction was allowed to proceed for a defined period, after which the glycerol microdroplets were collected, and the products were extracted by using ethyl acetate and purified using preparative thin-layer chromatography. Product analysis was conducted using ^1^H nuclear magnetic resonance (NMR) spectroscopy, and the conversion of 4-nitrobenzaldehyde was calculated based on the isolated yield:

Yield (%) = (mass of purified product) / (theoretical mass of product) × 100 (S1)

All liquid-state NMR spectra were recorded on a Bruker AVANCE III 500 spectrometer (500 MHz) by using deuterated dimethyl sulfoxide (*d*-DMSO) as the solvent. Reactions with various aldehydes were performed under similar conditions.

To investigate the role of chemical feeding in our system, two additional control experiments were conducted. In the first control, a 1-µL glycerol microdroplet containing both reactants (1 mmol 4-nitrobenzaldehyde and 2.5 mmol 2-methylindole) was placed directly on the pure LCIPS without stirring. In the second control, a larger 10-µL glycerol microdroplet was used on a nematic LCIPS pre-loaded with 2.5 mmol of 2-methylindole. Both control experiments were conducted at a consistent temperature of 90°C to maintain comparable reaction conditions. Product analysis and conversion calculations were performed as described above.

**Characterization of particle diffusion under chemical release**

To quantify the impact of chemical release on mass transfer, we tracked the motion of tracer particles in a glycerol/water film in contact with an LC film. The glycerol solution was prepared by mixing glycerol (49 vol%), water (49 vol%), and an aqueous dispersion of 1 µm-in-diameter polystyrene microspheres (2 vol%; particle content: 1% in the dispersion). To create LC–glycerol interfaces, optical cells were assembled by sandwiching 10 µL of the above glycerol/water particle dispersion and 10 µL of E7, with or without encapsulated glycerol microdroplets, between two DMOAP-functionalized glass slides spaced 0.5 mm apart. For chemical release experiments, the LC phase consisted of E7 (90 vol%) with encapsulated glycerol microdroplets (10 vol%; glycerol/water: 50/50 vol%). To prevent coalescence of the microdroplets within bulk E7, 5 mM SDS was added. The assembled cells were allowed to equilibrate for at least 30 min before measurement.

Tracer particle motion was recorded using an Olympus polarized light microscope at 20× magnification over 160 s, capturing frames every 2.13 s. The optical cells were heated to 70°C using a Linkam PE120 heating stage to induce chemical release. Particle trajectories were analyzed using the MTrackJ plug-in in ImageJ^[1]^. This software enabled the extraction of x- and y-coordinates over time, allowing quantification of diffusion coefficients under different conditions.

**Two-step cascade reaction within a single glycerol droplet microreactor achieved by spatially patterned chemical loading on LCIPS**

To demonstrate the unique capability of LCIPS for programmable, multi-step microscale synthesis, we performed a two-step cascade reaction within a single glycerol droplet microreactor using spatially patterned chemical loading (Fig. 6 of the main text). As a model system, we employed a thiol–ene coupling followed by oxidation. Step 1: A 1-µL glycerol microdroplet containing 1 mmol styrene was placed on Zone 1 of LCIPS. Upon heating to 90 °C, a localized nematic-to-isotropic phase transition released pre-loaded *p*-toluenethiol (2.5 mmol/cm^2^), initiating the thiol–ene coupling reaction. After the desired reaction time, the droplet was collected, products were extracted with ethyl acetate, and purified via preparative thin-layer chromatography. Step 2: The same droplet was then guided to Zone 2 of the LCIPS by tilting the substrate. A second local phase transition released pre-loaded H_2_O_2_ (2.5 mmol/cm^2^), oxidizing phenethyl(*p*-tolyl)sulfane to 1-methyl-4-(phenethylsulfinyl)benzene. Products were characterized by NMR spectroscopy, and yields were calculated according to Equation S1.

**Statistical analysis**

All averages were numerical averages, and all confidence intervals were calculated from samples sizes (*n*) = 3.

**Results and Discussion**

**Stability of LCIPS against glycerol-induced dewetting**

We comment here that LCIPS is stable against glycerol-induced dewetting. To this end, the total interfacial energy of the glycerol–wetted porous polyRM257 substrate (*E*_A_) must be higher than that wetted by the LC with (*E*_1_) or without (*E*_2_) a glycerol droplet on the surface. This can be written as^[2]^:

Δ*E*_1_ = *E*_A_ – *E*_1_ = *r* (*γ*_LC_ cos*θ*_LC_ – *γ*_Gly_ cos*θ*_Gly_) – *γ*_Gly–LC_ > 0 (S2)

Δ*E*_2_ = *E*_A_ – *E*_2_ = *r* (*γ*_LC_ cos*θ*_LC_ – *γ*_Gly_ cos*θ*_Gly_) + *γ*_Gly_ – *γ*_LC_ > 0 (S3)

in which *r* is the roughness factor (or the ratio of the true versus the projected surface areas of the porous surface), *γ*_g–LC_ is the interfacial tension between glycerol and the LC film, *γ*_g_ and *γ*_LC_ represent the surface tension of glycerol and the LC, respectively, and *θ*_g_ and *θ*_LC_ are the equilibrium contact angles between the glycerol and the LC on a solid surface, respectively (as mentioned below and summarized in Table S1). Using the values from Supplementary Table 1 and substituting them into Equations S2 and S3, the interfacial energies were calculated to be Δ*E*_1_ = +1.77 mJ/m^2^ and Δ*E*_2_ = +20.47 mJ/m^2^ at room temperature (25^o^C). These values remained positive even when *r* = 1, corresponding to flat surfaces. For reference, our porous polyRM257 substrates have *r* > 1. These results imply that the LC will be stable on the porous polyRM257 substrate through surface tension-induced capillary forces and will resist glycerol-induced dewetting.

**Estimation of LC wrapping layer at air–glycerol interface on droplets**

To explore the presence of a wrapping layer composed of nematic E7 on the glycerol droplets on a nematic LCIPS, we calculated the spreading coefficient (*S*) of nematic E7 at the air–glycerol interface using the following equation^[3]^:

*S* = *γ*_Gly_ – (*γ*_Gly–LC_ + *γ*_LC_) (S4)

where *γ*_Gly–LC_ is the interfacial tension between glycerol and the LC, and *γ*_Gly_ and *γ*_LC_ represent the surface tensions of glycerol and the LC, respectively. When *S* > 0, a wrapping layer is formed^[4]^. Using the data in Table S1 (*γ*_Gly_ = 47.6 mN/m, *γ*_Gly–LC_ = 5.6 mN/m, and *γ*_LC_ = 34.5 mN/m), we found *S* to be +7.5 mN/m, which confirms the presence of a nematic E7 wrapping layer around glycerol droplets on a nematic LC surface.

Next, we estimated the wrapping layer’s equilibrium thickness (*B*_t_) by combining and equating the disjoining pressure, which originates from van der Waals forces between molecules at the glycerol–LC and air–LC interfaces^[5]^:

*П*_disjoining_ = *A*_air–LC–Gly_/ 6π*B*_t_^3^ (S5)

where *A*_Air–LC–Gly_ is the Hamaker constant for the interaction between air and glycerol across the LC, and the capillary pressure, which arises due to the surface tension at the curved surface of the droplet (2*γ*_LC_ / *R*_Gly reactor_, where *R*_Gly reactor_ is the base radius of the glycerol droplet microreactor). *B*_t_ can be calculated using^[6]^:

*B*_t_ = $\left( \frac{\text{A}_{\text{A}\text{ir–LC–}\text{G}\text{ly}}R_{\text{Gly}\text{ reactor}}}{\text{12π}\text{γ}_{\text{LC}}} \right)$^1/3^ (S6)

*A*_Air–LC–Gly_ was set to 10^–20^ J, which is typical for organics interacting with glycerol and involving hydrogen bonding^[7]^. Equation S6 was used to calculate a *B*_t_ of ~ 20 nm for a 10-µL glycerol droplet (*R*_Gly reactor_ ~ 1.8 mm), which is on the same order of magnitude as the thickness of the wrapping layer of isotropic oils on glycerol droplets^[6b, 8]^. The thickness of the wrapping layer is much smaller than the LC film underneath the droplet so we can neglect the mass loss of LC lubricant associated with removal of glycerol droplets occurs mainly due to the removal of wetting ridges (rather than the wrapping layers) of the glycerol droplet. This result is consistent with previous reports on isotropic oil-based slippery lubricant-infused porous surface (SLIPS) systems^[6b, 8]^.

**Investigation of glycerol miscibility with isotropic and nematic phases of E7**

To investigate the miscibility of glycerol with isotropic phase LCs, we combined 0.25 mL of glycerol with 0.25 mL of E7. The mixture was heated to 90°C and maintained at this temperature for 10 min before allowing it to cool to room temperature. We observed that glycerol and isotropic E7 have a less distinct interface, indicating partial miscibility. However, upon cooling to the nematic phase, the solubility of glycerol in the LC significantly decreases, resulting in a clear phase separation between glycerol and the nematic phase LC. This phenomenon leads to the formation of micrometer-sized glycerol droplets within the bulk LC, with a notably distinct interface (Figure S2). This result is consistent with previous investigations that glycerol is partially miscible with isotropic phase LCs such as E7 and 4-cyano-4’-pentylbiphenyl (5CB)^[6b, 8]^. Building on this finding, we sought to use glycerol to achieve *in-situ* loading and release of chemicals within the LCIPS, potentially offering a more versatile and efficient approach to droplet microreactor systems.

**Fluorescence microscopy characterization of chemical loading and release process on LCIPS**

To elucidate the chemical loading and release processes on LCIPS, fluorescence microscopy experiments were conducted (Figure S3). A 10-µL droplet of RhB-doped glycerol was deposited onto an isotropic E7 film heated to 65°C for 5 min. Fluorescence micrographs revealed a uniform red coloration in the E7 film beneath the glycerol droplet, confirming effective molecular dispersion of RhB and glycerol. Upon cooling the LCIPS to the nematic phase, distinct red droplets with an average diameter of 2.0 ± 0.5 µm emerged, indicating phase separation of RhB-doped glycerol from the nematic E7 matrix. Subsequently, a 10-µL droplet of pure glycerol was placed on the nematic E7 film containing the RhB-doped microdroplets. Following the activated release of chemicals into the glycerol droplet via nematic–isotropic LC phase transition, fluorescence microscopy showed a significant reduction in the density of RhB-doped glycerol microdroplets within the LCIPS. This observation demonstrated that glycerol’s phase-specific solubility enables efficient *in-situ* chemical loading and release.

**Reusability of LCIPS for chemical loading and release**

The reusability of LCIPS was evaluated through multiple cycles of *in-situ* loading and release. Initially, a 10-µL droplet of RhB-doped glycerol was absorbed into an isotropic E7 film at 65°C for 5 min. Upon cooling the film to the nematic phase, distinct red droplets with an average diameter of 2.0 ± 0.5 µm formed, reflecting phase separation. A subsequent 10-µL droplet of pure glycerol placed on the nematic E7 film did not exhibit measurable release for at least 20 min, underscoring the stability of the nematic phase.

Upon heating to induce a transition to the isotropic phase, continuous release of RhB-doped glycerol into the overlying glycerol droplet was observed. Approximately 2.48 mg of encapsulated RhB-doped glycerol was released within 60 min. Notably, this process was repeatable for at least 10 cycles without significant efficiency loss (Figure 2D, main text), highlighting the robustness and long-term applicability of LCIPS for chemical loading and release.

**Thermodynamic model for phase-dependent chemical release in LCIPS**

In the main text, we observed that the nematic-to-isotropic transition results in chemical release of glycerol microdroplets to the glycerol droplet microreactor at the LC surface. In this section, we describe interactions in the thermodynamic model to understand this phase-dependent phase behavior.

The attractive van der Waals force (*F*_vdW_) between the glycerol microdroplet and the glycerol droplet microreactor can be written as:^[7a]^

$F_{\text{vdW}}=-\frac{\text{A}_{\text{Gly–LC–Gly}}R_{\text{Gly}\text{ mdrop}}R_{\text{Gly}\text{ reactor}}}{6\text{x}^{2}(R_{\text{Gly mdrop}}+R_{\text{Gly reactor}})}$ (S7)

where *A*_Gly–LC–Gly_ is the Hamaker constant for the interaction between two glycerol phases across the LC, *R*_Gly mdrop_ is the radius of the encapsulated glycerol microdroplet, and *x* is the surface-to-surface distance between the glycerol microdroplet and glycerol droplet microreactor on the LCIPS. For our calculations, we assumed *A*_Gly–LC–Gly_ = 1 × 10^–20^ J (typical value for organics interacting with water or polar solvents involving hydrogen bonding),^[7]^ *R*_Gly mdrop_ = 5 µm and *R*_Gly reactor_ = 1.8 mm. As *R*_Gly reactor_ >> *R*_Gly mdrop_, we can simplify Equation S7 to:

$F_{\text{vdW}}=-\frac{\text{A}_{\text{Gly–LC–Gly}}R_{\text{Gly}\text{ mdrop}}}{6\text{x}^{2}}$ (S8)

Equation S8 is found to be negative. This is indicative of the attractive nature of *F*_vdW_ in the process of releasing encapsulated glycerol microdroplets.

When placing a glycerol droplet microreactor on the surface of the LCIPS, a wetting ridge forms on the LC surface that encompasses the droplet. This meniscus surrounding the droplet has a capillary force (*F*_cap_) that concentrates the encapsulated glycerol microdroplets into the wetting ridge. To understand the capillary force’s impact in the chemical release process, we derive an expression for the estimation of the capillary force.

Along any point in a curved LC surface, the local hydrostatic pressure *P* can be found from the Laplace pressure:^[9]^

$P= P_{\text{atm}}+\gamma_{\text{LC}}\left( -\frac{1}{R_{\text{1}}}+\frac{1}{R_{\text{2}}} \right)$ (S9)

where *P*_atm_ is atmospheric pressure, and *R*_1_ and *R*_2_ are the two principal radii of curvature. The positive and negative signs relate directly to the positive and negative curvature of *R*_1_ and *R*_2_, respectively. From Equation S9, the hydrostatic pressure in the wetting ridge (*P*_ridge_) can be written as:

$P_{\text{ridge}}\approx P_{\text{atm}}+\gamma_{\text{LC}}\left( -\frac{1}{R_{\text{1}}}+\frac{1}{R_{\text{Gly}\text{ reactor}}} \right)$ (S10)

In our system, *R*_1_ < *R*_Gly reactor_, suggesting *P*_atm_ > *P*_ridge_.^[4]^ This indicates the capillary force induced by the hydrostatic pressure gradient within the LC wetting ridge supplies a driving force for the glycerol microdroplets to concentrate into the wetting ridge around the glycerol droplet microreactor at the LC surface. This characteristic capillary length where the capillary force is similar to the gravitational force is shown as:^[9]^

$\text{capillary length}= \sqrt{{\gamma_{\text{LC}}}/\left( \rho_{\text{LC}}g \right)}$ (S11)

in which *ρ*_LC_ is the mass density of the LC (1.02 g/cm^3^).^[10]^ We can estimate the capillary length to be approximately 1.8 mm. Any glycerol microdroplets within 1.8 mm of the glycerol droplet microreactor are effectively attracted to its wetting ridge.

The curved air–LC surface causes a static pressure jump across the glycerol microdroplets. The average hydrostatic pressures experienced by the hemispheres of glycerol microdroplets encapsulated inside to the curved LC surface toward and away from the glycerol droplet microreactor can be written as:

$P_{\text{Gly}\text{ mdrop}, 1}\approx P_{\text{atm}}+\gamma_{\text{LC}}\left( -\frac{1}{R_{\text{1}}}+\frac{1}{R_{\text{Gly}\text{ reactor}}+x-\xi R_{\text{Gly}\text{ mdrop}}} \right)$ (S12)

$P_{\text{Gly}\text{ mdrop}, 2}\approx P_{\text{atm}}+\gamma_{\text{LC}}\left( -\frac{1}{R_{\text{1}}}+\frac{1}{R_{\text{Gly}\text{ reactor}}+x+\xi R_{\text{Gly}\text{ mdrop}}} \right)$ (S13)

where *ξ* is a coefficient that is used to estimate the average hydrostatic pressure on each interacting hemisphere (*ξ* = 2/3).^[7a]^

The hydrostatic pressure gradient (Δ*P*) around the glycerol microdroplets can be written as:

$\Delta P=P_{\text{Gly}\text{ mdrop}, 2}- P_{\text{Gly}\text{ mdrop},1}$

$\approx\gamma_{\text{LC}}\left( \frac{1}{R_{\text{Gly}\text{ reactor}}+x+\xi R_{\text{Gly}\text{ mdrop}}}-\frac{1}{R_{\text{Gly}\text{ reactor}}+x-{\xi R}_{\text{Gly}\text{ mdrop}}} \right)$

$= -\frac{2\text{γ}_{\text{LC}}\text{ξ}R_{\text{Gly}\text{ mdrop}}}{\left( R_{\text{Gly}\text{ reactor}}+\text{x} \right)^{2}-\left( {\xi R}_{\text{Gly}\text{ mdrop}} \right)^{2}}$

$\approx-\frac{2\text{γ}_{\text{LC}}\text{ξ}R_{\text{Gly}\text{ mdrop}}^{3}}{\left( R_{\text{Gly}\text{ reactor}}+\text{x} \right)^{2}} (\mathrm{for}R_{\text{Gly}\text{ mdrop}}\ll R_{\text{Gly}\text{ reactor}})$ (S14)

By multiplying Δ*P* by the cross-sectional area of the glycerol microdroplet, we have the capillary force (*F*_cap_):

$F_{\text{cap}}=\Delta P\pi R_{\text{Gly}\text{ mdrop}}^{2}\approx-\frac{2\text{γ}_{\text{LC}}\text{ξπ}R_{\text{Gly}\text{ mdrop}}^{3}}{\left( R_{\text{Gly}\text{ reactor}}+\text{x} \right)^{2}}$ (S15)

We highlight the negative sign of the *F*_cap_ indicating the attractive nature of this force that acts as the driving force for the chemical release.

When *R*_Gly reactor_ >> *R*_Gly mdrop_, the electric double layer force (*F*_edl_) between the glycerol microdroplet and glycerol droplet microreactor is described by:^[11]^

$F_{\text{edl}}\text{ = 64}\text{π}\text{ε}_{\text{LC}}\text{ε}_{\text{0}}{\text{(}\frac{k_{\text{B}}T}{e}\text{)}}^{\text{2}}\tanh\text{(}\frac{\text{ez}\text{ψ}_{\text{Gly}\text{ reactor}}}{\text{4}\text{k}_{\text{B}}\text{T}}\text{)tanh} \text{(}\frac{\text{ez}\text{ψ}_{\text{Gly}\text{ mdrop}}}{\text{4}\text{k}_{\text{B}}\text{T}}\text{)}\text{R}_{\text{Gly}\text{ mdrop}}\text{exp(}-\text{κx}\text{)}$ (S16)

where *ε*_LC_ is the relative permittivity (*ε*_LC_ = 10, the relativity of nematic E7),^[12]^ *ε*_0_ is the vacuum permittivity (*ε*_0_ = 8.854 × 10^–12^ C/V m), *k*_B_ is the Boltzmann constant (*k*_B_ = 1.38 × 10^–23^ J/K), *T* is the temperature (*T* = 298 K and 343 K for the nematic and isotropic phases, respectively), *z* is the valence number of the ionic species (*z* = 1 for both glycerol microdroplet and glycerol droplet microreactor), *e* is the elementary charge (*e* = 1.3 × 10^–19^ C), *κ*^–1^is the Debye length (*κ*^–1^ = 2 μm in the LC without added electrolytes),^[13]^ *ψ*_Gly reactor_ is the zeta potential of the glycerol droplet microreactors, and *ψ*_Gly mdrop_ is the zeta potential of the encapsulated glycerol microdroplets. *ψ*_Gly mdrop_ is set to –100 mV and *ψ*_Gly reactor_ is set to –20 mV.^[11]^ Depending on the phase, *F*_edl_ can be either attractive or repulsive. If *ψ*_Gly reactor_ and *ψ*_Gly mdrop_ are the same sign, *F*_edl_ is repulsive, while *F*_edl_ is attractive when the signs are opposite.

There are repulsive interactions caused by the long-range orientational ordering of the bulk LC when there are colloidal particles moving towards the surface.^[14]^ The elastic force from the LC (*F*_el_) between the glycerol microdroplet and glycerol droplet microreactor interface is found from:^[14b]^

$F_{\text{el}}=+ \frac{\text{π}\text{α}^{\text{2}}\text{βK}R_{\text{Gly}\text{ mdrop}}^{4}}{{(R_{\text{Gly}\text{ mdrop}}+\text{x})}^{4}}$ (S17)

where *α* and *β* represent the dipole and quadrupole moments of the glycerol microdroplets in LC and *K* is the Frank elastic constant of the LC. *α* is 2.04 for the glycerol microdroplet with homeotropic anchoring in nematic phase,^[14b]^ and 0 for glycerol microdroplets in isotropic LCs. *β* = 1/2 for homeotropic anchoring.^[11]^ *K* is set at 5 pN.^[15]^ The positive sign indicates the *F*_el_ is always repulsive and impedes the release of glycerol microdroplets.

The combination of the previous forces into a single thermodynamic model describes the key features of the activated release behavior at LC surfaces. The net force (*F*_net_) acting on the glycerol microdroplets in the LC is represented as:

$\text{F}_{\text{net}}\text{ = }\text{F}_{\text{vdW}}\text{ + }\text{F}_{\text{cap}}\text{ + }\text{F}_{\text{edl}}\text{ + }\text{F}_{\begin{aligned} \text{el} \\ \text{ } \end{aligned}}=$

$-\frac{\text{A}_{\text{Gly–LC–Gly}}R_{\text{Gly}\text{ mdrop}}}{6\text{x}^{2}}-\frac{2\text{γ}_{\text{LC}}\text{ξπ}R_{\text{Gly}\text{ mdrop}}^{3}}{\left( \text{R}_{\text{Gly reactor}}+\text{x} \right)^{2}}+$

$\text{64}\text{π}\text{ε}_{\text{LC}}\text{ε}_{\text{0}}{\text{(}\frac{k_{B}T}{e}\text{)}}^{\text{2}}\tanh\text{(}\frac{\text{ez}\text{ψ}_{\text{Gly reactor}}}{\text{4}\text{k}_{\text{B}}\text{T}}\text{)tanh} \text{(}\frac{\text{ez}\text{ψ}_{\text{Gly}\text{ mdrop}}}{\text{4}\text{k}_{\text{B}}\text{T}}\text{)}\text{R}_{\text{Gly}\text{ mdrop}}\text{exp(}-\text{κx}\text{)}+\frac{\text{π}\text{α}^{\text{2}}\text{βK}R_{\text{Gly}\text{ mdrop}}^{4}}{\left( R_{\text{Gly}\text{ mdrop}}+\text{x} \right)^{4}}$ (S18)

Equation S18 is identical to Equation 1 in the main text depicting the nematic-to-isotropic transition, induced by heating, initiating the release of the glycerol microdroplets from the bulk LC to the glycerol droplet microreactor. We calculated the *F*_net_ on the glycerol microdroplets in the nematic LC surface where the glycerol droplet microreactor has been placed. It was found that the repulsive forces, *F*_edl_ and *F*_el_, created a kinetic barrier, preventing the release of glycerol microdroplets consistent with previous experiments.

To test the phase-dependence, we calculated *F*_net_ on the glycerol microdroplets in the isotropic LC surface where the glycerol droplet microreactor has been placed. In the isotropic phase, *F*_el_ is set to be zero. The absence of the repulsive *F*_el_ force causes *F*_net_ to become an attractive force (negative), overcoming the repulsive forces and releasing the glycerol microdroplets into the glycerol droplet microreactor. The thermodynamic model has proven the nematic-to-isotropic transition induced by heat activated the release of glycerol microdroplets within the LC surface and confirming the importance of the orientation order in the release behavior.

**Degradation and removal of organics in glycerol droplet reactors by LCIPS**

In addition to the chemical reactions occurring in the droplet reactors, we also investigate the degradation and removal of organic pollution in glycerol droplet reactors using LCIPS. RhB was selected as a model organic pollutant (Figure S8). Two glycerol droplets, one containing 1 wt% TiO_2_ particles and the other without, were placed on nematic E7 films. After the thermally triggered release of RhB (~2 mg), only the droplet containing TiO_2_ particles became colorless, indicating effective TiO_2_-mediated photocatalytic degradation. In contrast, the RhB-doped glycerol droplet without TiO_2_ remained red.

UV–visible absorbance measurements and reaction kinetics further confirmed the effective degradation and removal of RhB in TiO_2_-containing glycerol droplet reactors on LCIPS. Reusability tests of the TiO_2_-mediated photocatalytic degradation demonstrated that the LCIPS system maintained consistent performance over multiple cycles, emphasizing its potential for practical applications in organic pollutant remediation.

**Quantification of particle diffusion in glycerol**

To quantify the effect of chemical release on particle motion, we analyzed the diffusion behavior of tracer particles in glycerol under two conditions: with and without glycerol microdroplet release (Figure 4a). We tracked the trajectories of 10 individual particles (5 in the release condition and 5 in the no-release condition) while heating the system to 70°C. Prior to the LC nematic-to-isotropic phase transition, particle motion in both cases was comparable, exhibiting behavior consistent with Brownian motion. However, upon phase transition, the release of glycerol microdroplets induced convective flow, leading to a significant increase in particle displacement in the release condition.

To quantify this effect, we calculated the time-dependent diffusion coefficients for each particle. The two-dimensional mean squared displacement (MSD) was determined using:

$MSD=4Dt$ (S19)

where *D* is the diffusion coefficient and *t* is the time. The MSD at each time step was computed using particle coordinates $\left( x_{t-\Delta t}, y_{t-\Delta t} \right)$ and $\left( x_{t+\Delta t}, y_{t+\Delta t} \right)$, given by:

$MSD=\left( x_{t+\Delta t}-x_{t-\Delta t} \right)^{2}+\left( y_{t+\Delta t}-y_{t-\Delta t} \right)^{2}$ (S20)

By substituting Equation S20 into Equation S19, we obtained the instantaneous diffusion coefficient:

$D\left( t \right)=\frac{\left( x_{t+\Delta t}-x_{t-\Delta t} \right)^{2}+\left( y_{t+\Delta t}-y_{t-\Delta t} \right)^{2}}{4\times(2\Delta t)}$ (S21)

Tracking *D*(t) over time revealed that before the phase transition, both systems exhibited similar diffusion coefficients consistent with Brownian motion. After the transition, the release of glycerol microdroplets induced convective flow, resulting in a pronounced increase in the instantaneous diffusion coefficient. This confirmed the role of chemical release in enhancing mass transfer within the glycerol phase.

**Table S1.** Interfacial tensions of air–glycerol, glycerol–LC, and air–LC interfaces and contact angle measurements of glycerol and LCs on porous substrates. *n*=3 for the means and standard deviations.

| *γ*_Gly_ (mN/m) | 47.6 ± 0.6 |
| --- | --- |
| *γ*_Gly–LC_ (mN/m) | 5.6 ± 1.6 |
| *γ*_LC_ (mN/m) | 34.5 ± 1.7 |
| *θ*_Gly_ (^o^) | 55.1 ± 0.5 |
| *θ*_LC_ (^o^) | 0 |


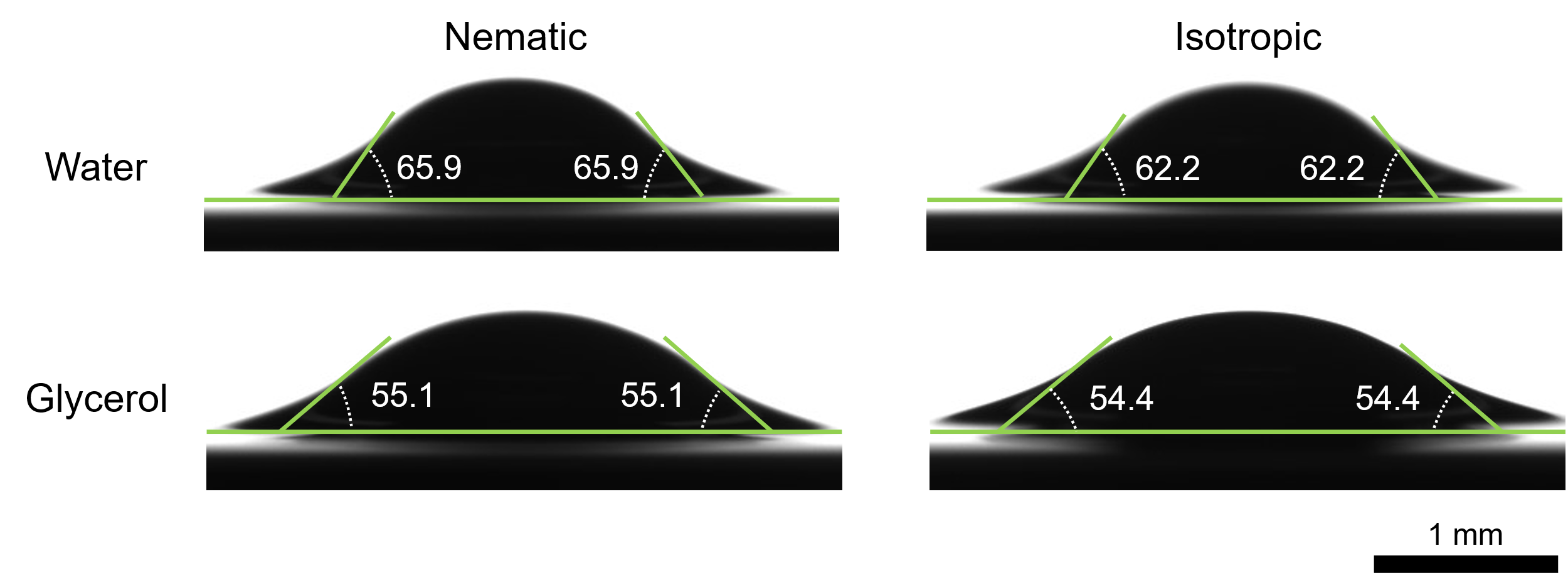


**Figure S1.** Apparent advancing contact angle *θ*_adv_ of glycerol and water droplets on LCIPS. Micrographs showing *θ*_adv_ on E7 surfaces in various phases. The micrographs were captured at 25^o^C and 65^o^C, corresponding to nematic and isotropic phases, respectively. The volumes of all droplets were 3 µL.


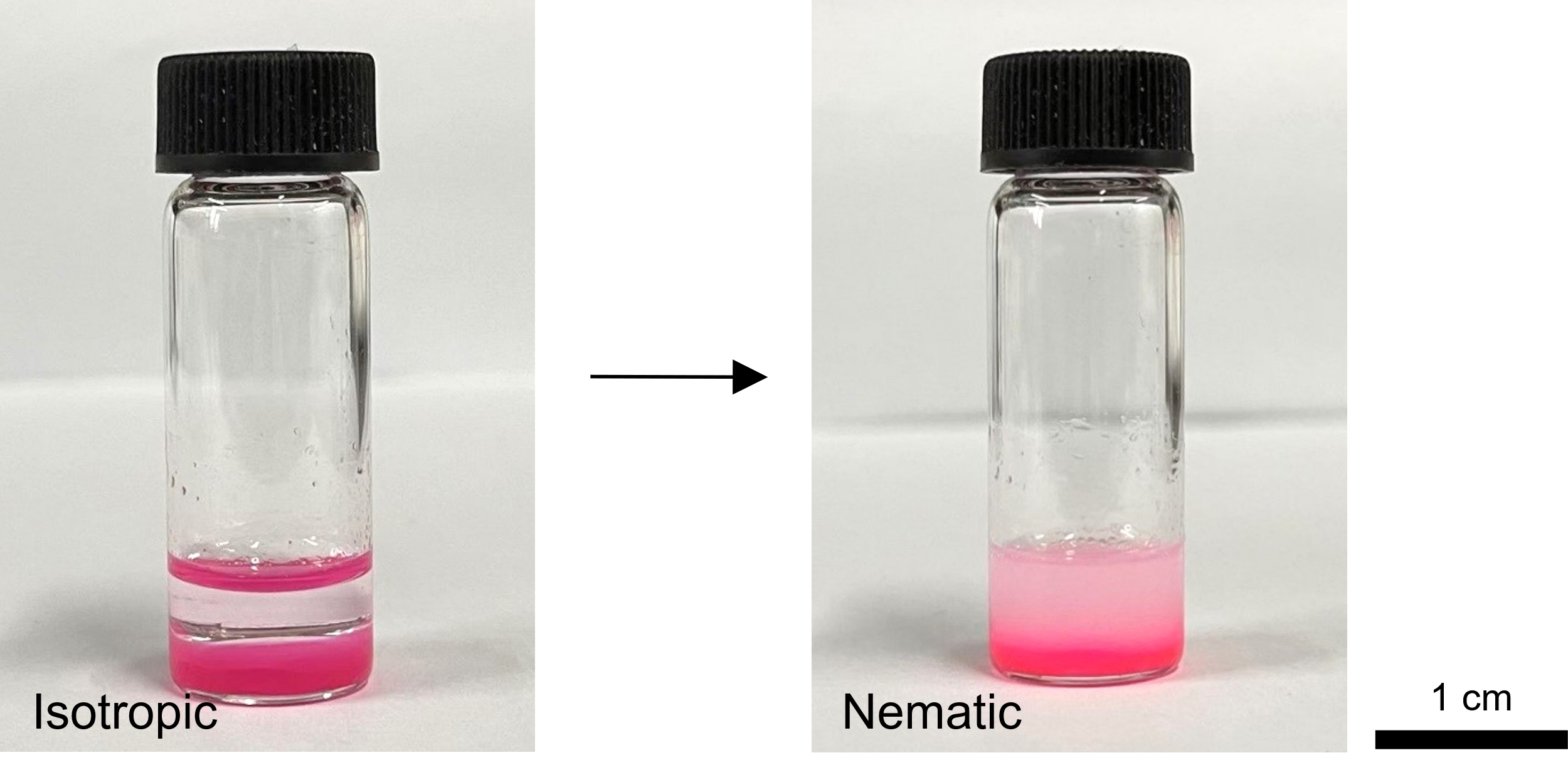


**Figure S2.** Miscible test of glycerol with isotropic phase E7. Photographs showing that RhB-loaded glycerol mixed with E7 can partially be miscible with isotropic phase E7. The solubility of glycerol in the nematic phase E7 significantly decreases, resulting in phase separation between glycerol and the nematic phase E7. The volumes of RhB-loaded glycerol and E7 were 0.5 mL. The concentration of RhB in the glycerol solution was 1 mg/mL.


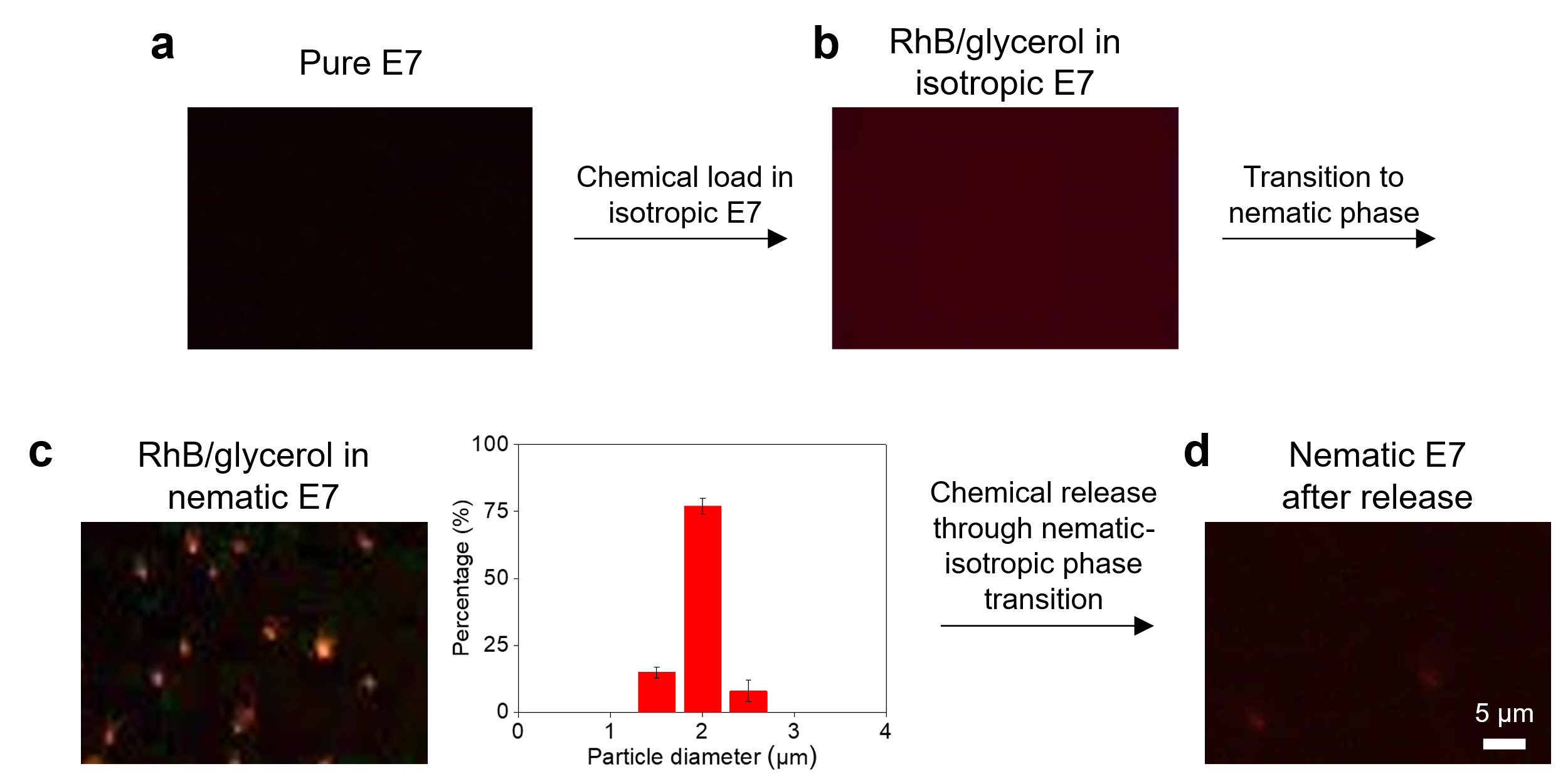


**Figure S3.** Fluorescence micrographs of chemical loading and release process on LCs. Fluorescent micrographs of LCIPS of (a) before and (b) after loading RhB-doped glycerol within the isotropic E7 film, (c) after subsequent transition into nematic phase, resulting in formation of RhB-loaded glycerol microdroplets in the bulk LC, and (d) after release of RhB-loaded glycerol microdroplets through LC phase transition. Inset plot in (c) shows the corresponding size distributions of the formed RhB-loaded glycerol microdroplets. Error bars represent standard deviations and *n*=3 for each data point.


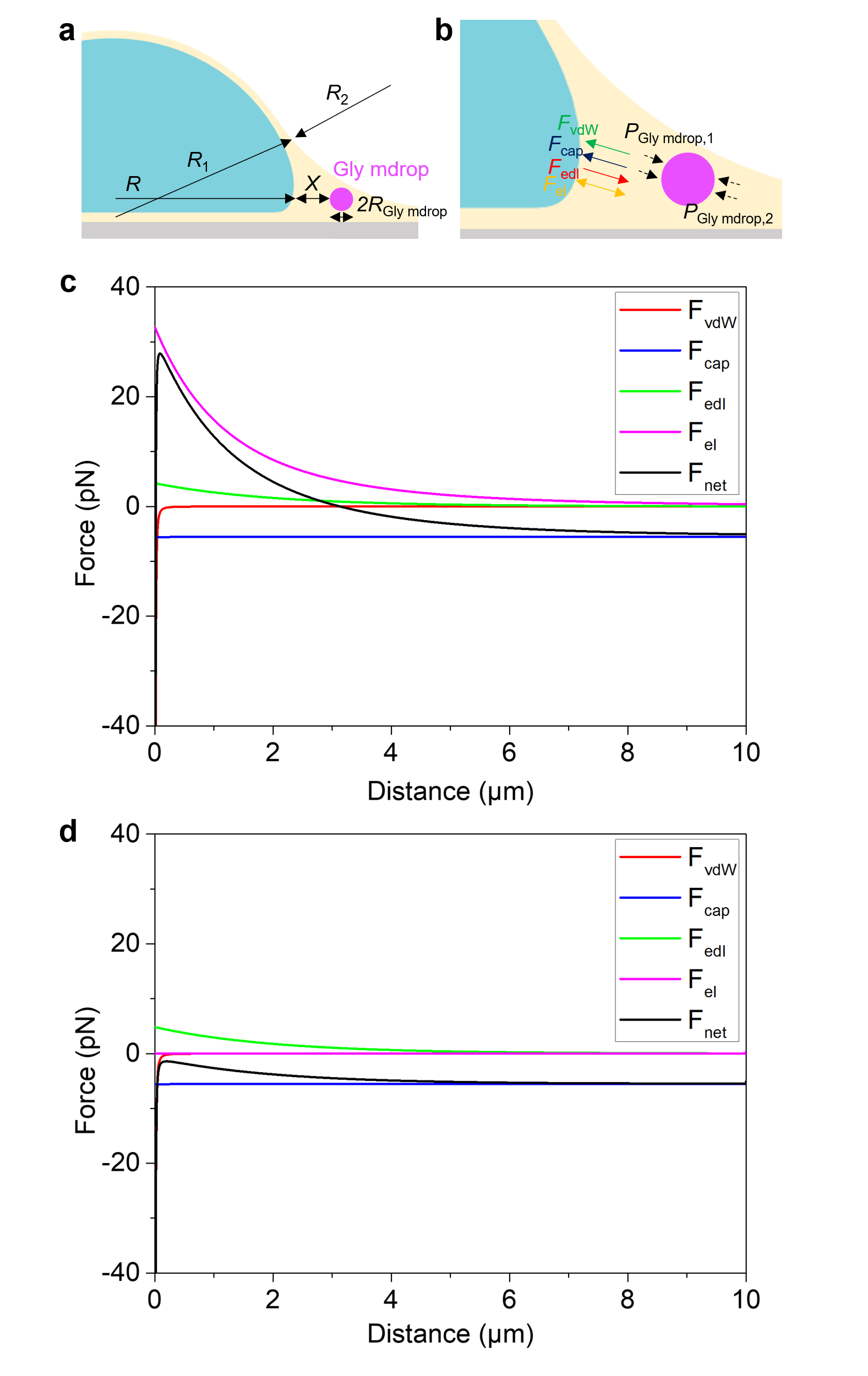


**Figure S4. Calculated interaction forces acting upon glycerol microdroplets.** (a) Schematic illustration (side view; not scaled to actual size) of interaction between the encapsulated glycerol microdroplet and glycerol droplet microreactor. *R*_1_ and *R*_2_ are the two principal radii of curvature. *R*_Gly reactor_ and *R*_Gly mdrop_ are the base radius of the glycerol droplet microreactor and the radius of the glycerol microdroplets dispersed in the LC film, respectively. *x* is the surface-to-surface distance between the glycerol microdroplet and glycerol droplet microreactor. (b) Schematic illustration (side view; not scaled to actual size) showing the hydrostatic pressures experienced by the hemispheres of glycerol microdroplets and all the interactions between glycerol microdroplets and glycerol droplet microreactor. *P*_Gly mdrop,2_ – *P*_Gly mdrop,1_ indicates the hydrostatic pressure gradient across the glycerol microdroplet. (c,d) Forces acting on glycerol microdroplets as a function of *x*, where the glycerol microdroplets are encapsulated in (c) nematic or (d) isotropic LC films.


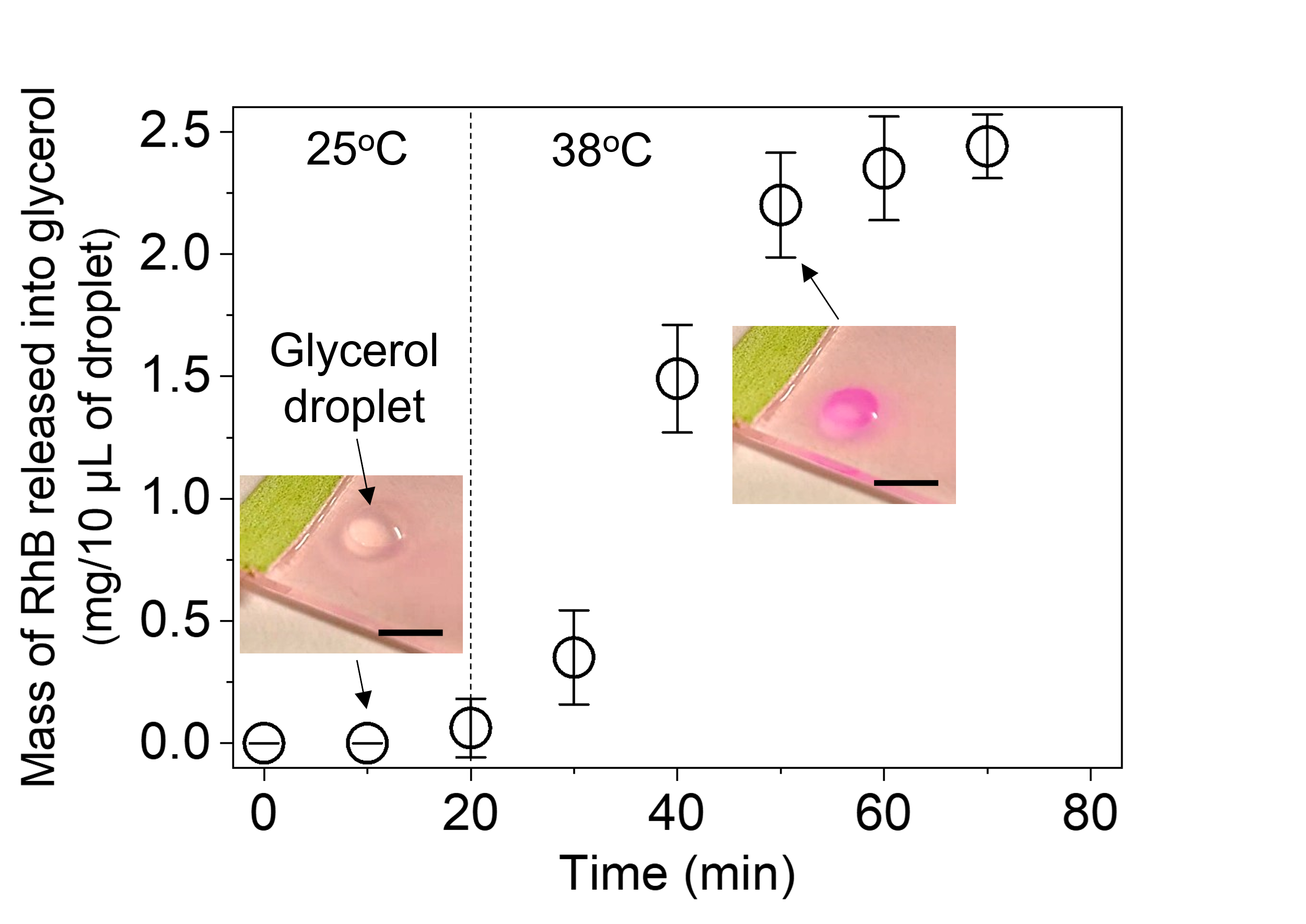


**Figure S5.** Chemical release from 5CB film. Plot showing the mass of RhB released into glycerol on LCIPS as a function of the time LCIPS was in the isotropic phase. The temperature was set to 25°C and 38°C to achieve nematic and isotropic phase of 5CB, respectively. Scale bars, 5 mm. Error bars represent standard deviations and *n*=3 for each data point.


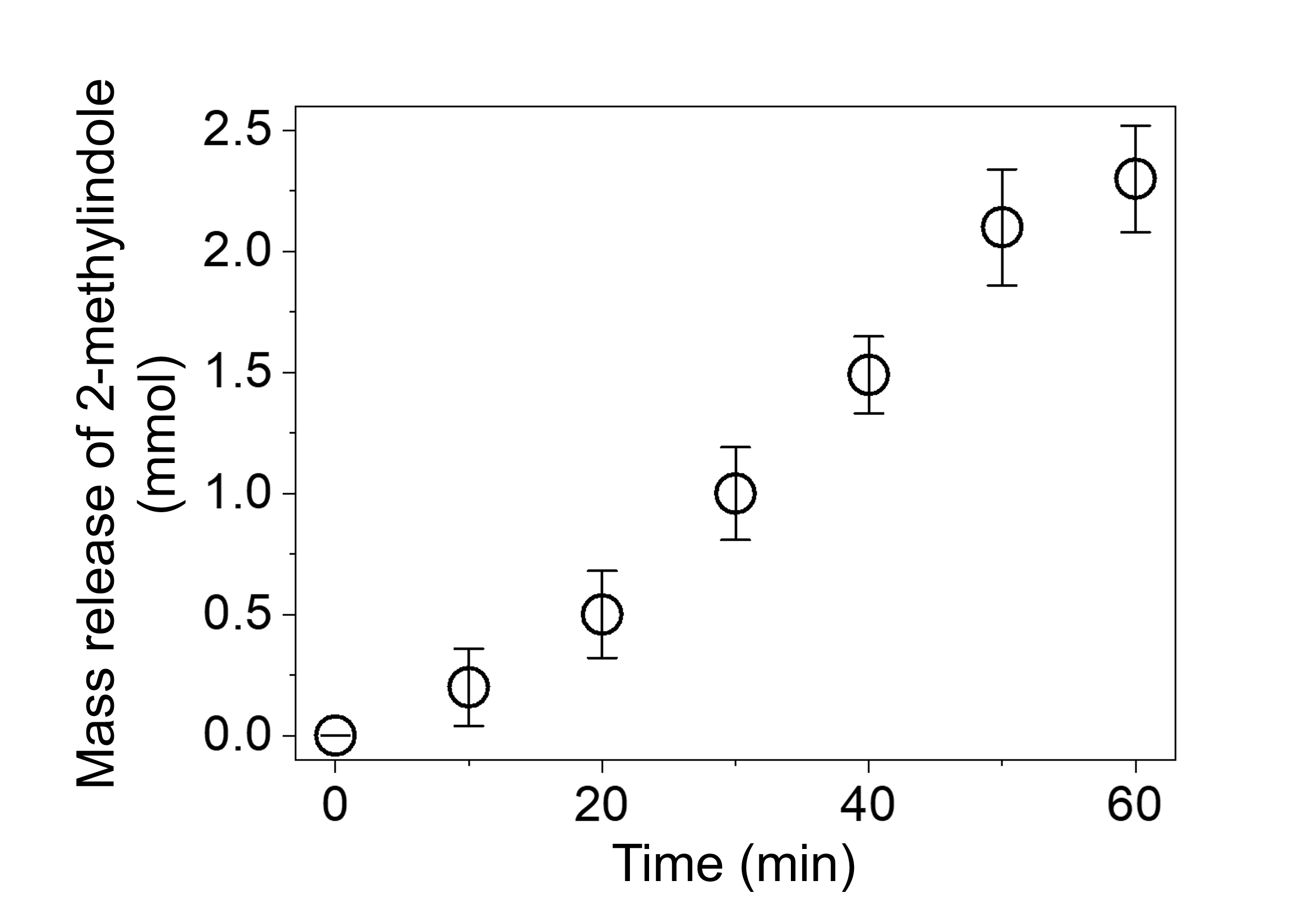


**Figure S6.** Mass of 2-methylindole released into glycerol on LCIPS as a function of time LCIPS in the isotropic phase. The initial amount of 2-methylindole in LCIPS was 2.5 mmol/cm^2^. Error bars represent standard deviations, with *n*=3 for each data point.


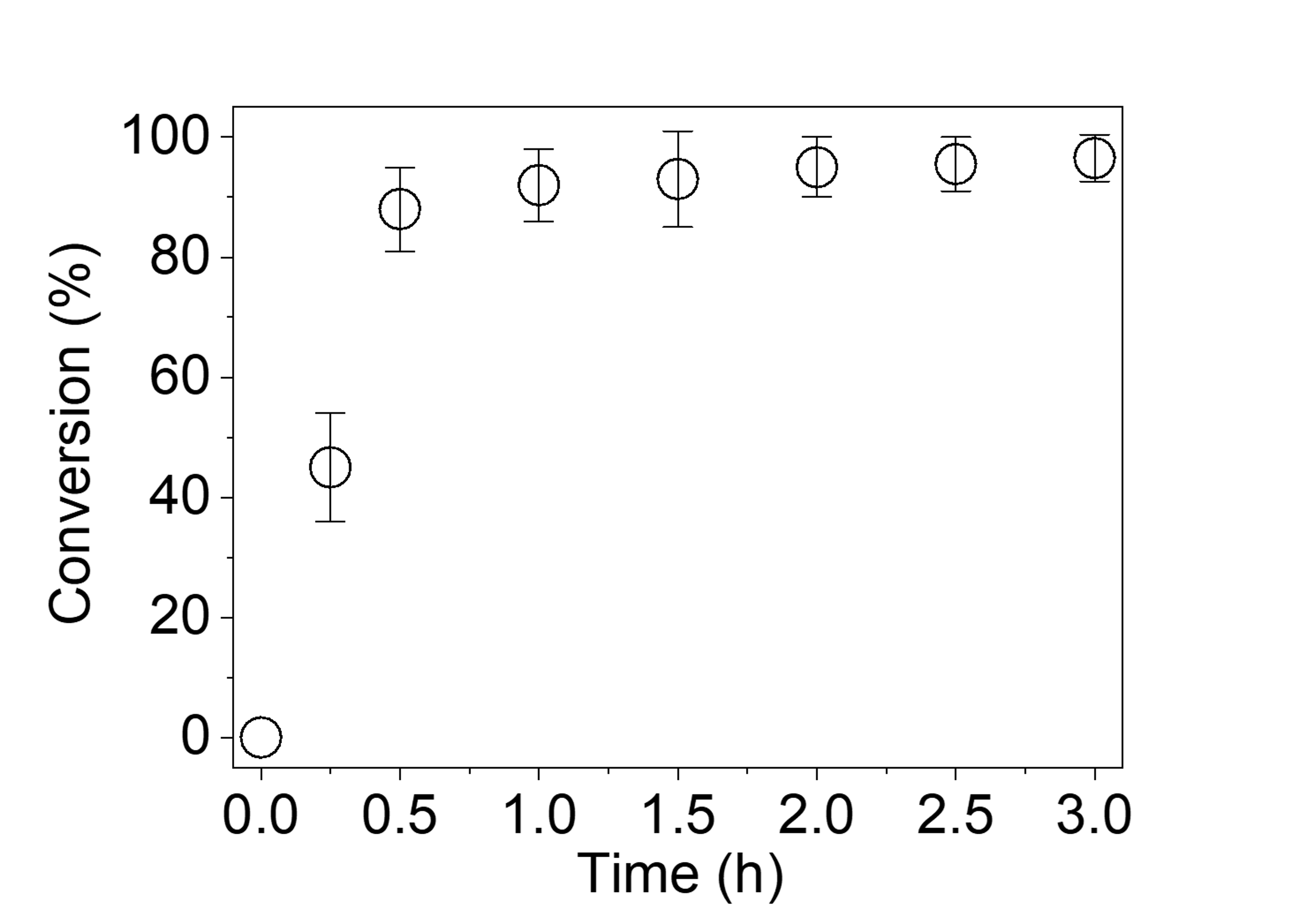


**Figure S7.** Effect of reagent addition sequence on reaction efficiency in droplet microreactors. Plot showing the conversion of 2-methylindole as aa function of time when a 1-µL glycerol microdroplet containing 2-methylindole was applied to an LCIPS loaded with 4-nitrobenzaldehyde. Reactions were initiated by heating to 90°C to trigger the nematic-to-isotropic phase transition. The initial amounts of 4-nitrobenzaldehyde and 2-methylindole in the LCIPS or glycerol droplet reactor were 2.5 mmol (per cm^2^ of LCIPS) and 1 mmol, respectively. Error bars represent standard deviations, with *n*=3 for each data point.


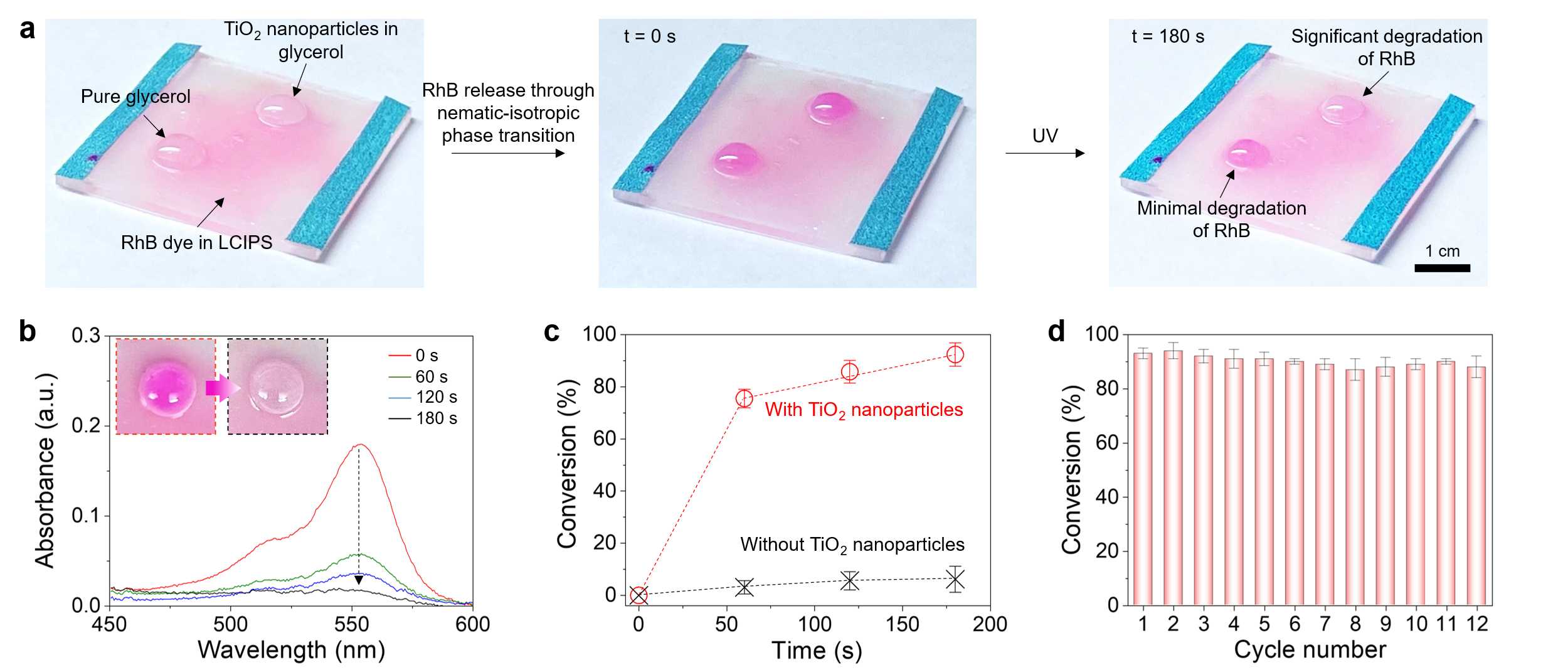


**Figure S8.** Degradation and removal of organics in glycerol droplet reactors by LCIPS. a) Sequential photographs showing thermal-triggered release of RhB and subsequent TiO_2_-mediated photocatalytic degradation of RhB-doped glycerol droplets; 1 wt% of TiO_2_ in droplet. Scale bars, 0.5 cm. b,c) Corresponding UV–visible absorbance (b) and reaction kinetics (c) of TiO_2_-mediated photocatalytic degradation of RhB at the E7 film in a. d) Plot showing the reusability of TiO_2_-loaded E7 film for RhB degradation. Error bars represent standard deviations and *n*=3 for each data point.


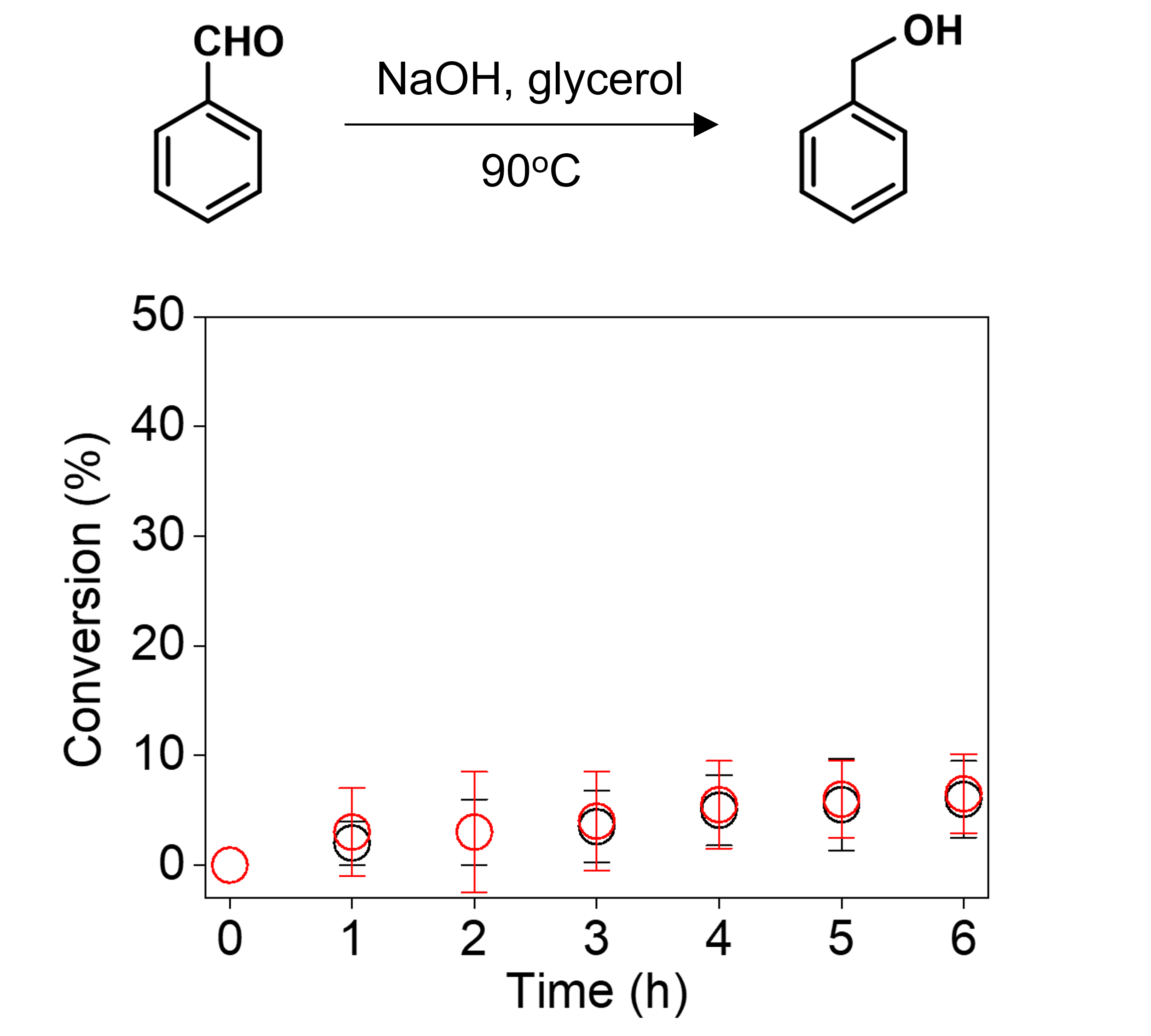


**Figure S9.** Effect of LC-mediated chemical feeding on a kinetically limited reaction. Conversion of benzaldehyde to benzyl alcohol over time for 1-µL droplet microreactors with (red) and without (black) LC-mediated chemical feeding. Red: 0.5-µL glycerol droplet containing 0.1 mmol benzaldehyde and 0.005 mmol sodium hydroxide on LCIPS loaded with 0.5-µL glycerol microdroplet; Black: 1-µL glycerol droplet containing 0.1 mmol benzaldehyde and 0.005 mmol sodium hydroxide on pure LCIPS. The chemical feeding and reactions were initiated by rapidly heating the system to 90°C. Error bars represent standard deviations, with *n*=3 for each data point.

**^1^H NMR data for compounds of condensation reaction products**

**3,3’-((4-Nitrophenyl)methylene)bis(2-methyl-1*H*-indole)**

^1^H NMR (500 MHz, *d*-DMSO): δ 10.86 (s, 2H); 8.15 (d, J = 1.5 Hz, 2H); 7.42 (d, J = 8 Hz, 4H); 7.24 (d, J = 8 Hz, 2H); 6.89 (t, J = 7 Hz, 2H); 6.79 (t, J = 9 Hz, 2H); 6.00 (s, 1 H); 2.11 (s, 6H).


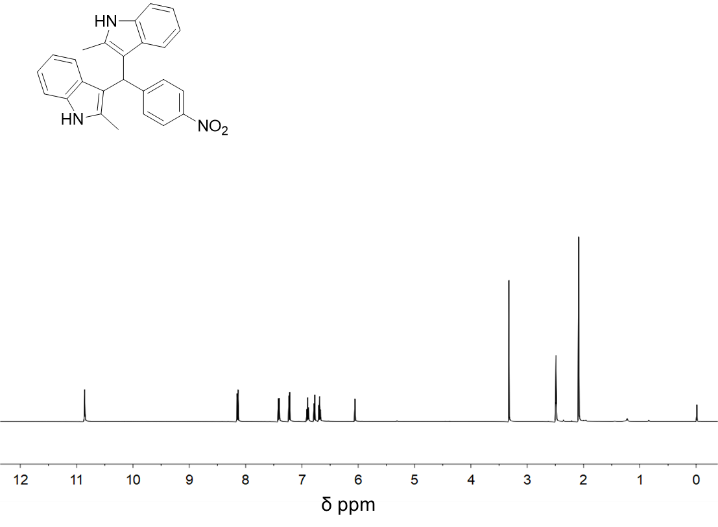


**3,3’-((2-Nitrophenyl)methylene)bis(2-methyl-1*H*-indole)**

^1^H NMR (500 MHz, *d*-DMSO): δ 10.84 (s, 2H); 7.83 (d, J = 8 Hz, 2H); 7.56 (t, J = 7.5 Hz, 4H); 7.49 (t, J = 7.5 Hz, 2H); 7.26 (d, J = 7.5 Hz, 2H); 6.90 (m, 2H); 6.57 (s, 1 H); 2.10 (s, 6H).


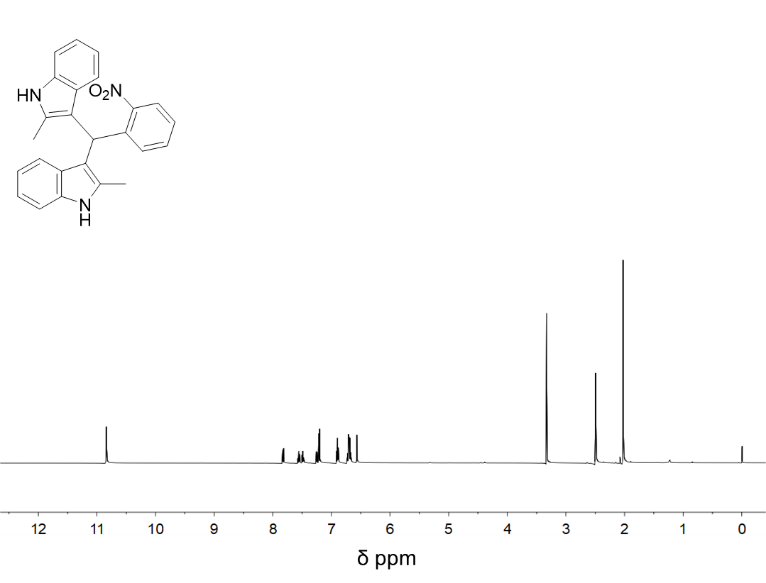


**3,3’-((3-Nitrophenyl)methylene)bis(2-methyl-1*H*-indole)**

^1^H NMR (500 MHz, *d*-DMSO): δ 10.86 (s, 2H); 8.10 (d, J = 8 Hz, 2H); 7.98 (t, J = 7.5 Hz, 4H); 7.56 (t, J = 8 Hz, 2H); 7.24 (d, J = 8 Hz, 2H); 6.79 (m, 2H); 6.10 (s, 1 H); 2.10 (s, 6H).


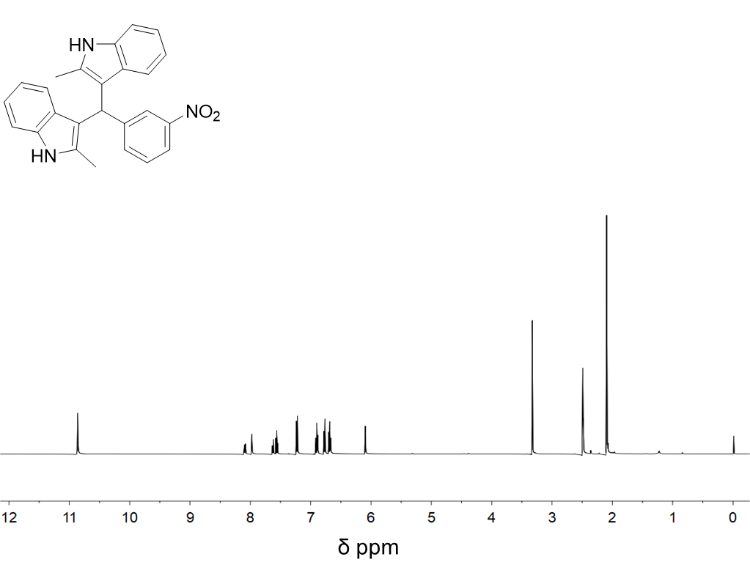


**3,3’-((4-Bromophenyl)methylene)bis(2-methyl-1H-indole)**

^1^H NMR (500 MHz, *d*-DMSO): δ 10.86 (s, 2H); 7.46 (d, J = 8 Hz, 2H); 7.23 (t, J = 7.5 Hz, 4H); 6.90 (t, J = 8 Hz, 2H); 6.80 (d, J = 8 Hz, 2H); 6.69 (t, J = 8 Hz, 2H); 5.90 (s, 1 H); 2.10 (s, 6H).


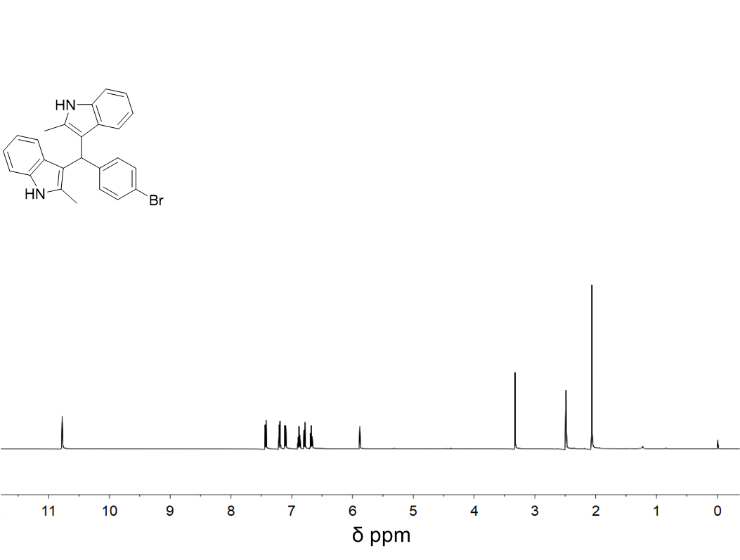


**3,3’-((4-Methoxyphenyl)methylene)bis(2-methyl-1*H*-indole)**

^1^H NMR (500 MHz, *d*-DMSO): δ 10.72 (s, 2H); 7.21 (d, J = 8 Hz, 2H); 7.09 (d, J = 8 Hz, 2H); 6.88 (t, J = 8 Hz, 2H); 6.83 (m, 4H); 6.68 (m, 2H); 5.85 (s, 1 H); 3.72 (s, 3 H); 2.10 (s, 6H).


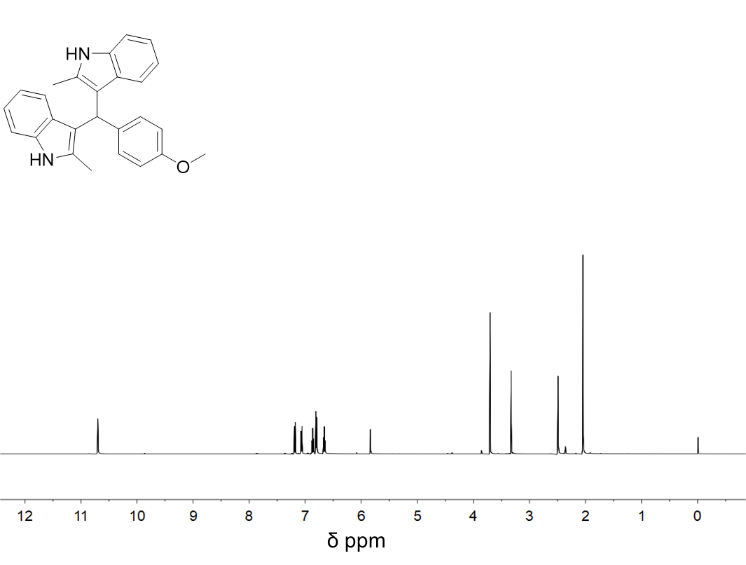


**3,3’-(p-Tolylmethylene)bis(2-methyl-1*H*-indole)**

^1^H NMR (500 MHz, *d*-DMSO): δ 10.70 (s, 2H); 7.19 (d, J = 8 Hz, 2H); 7.04 (d, J = 8 Hz, 2H); 6.89 (t, J = 8 Hz, 2H); 6.81 (m, 4H); 6.65 (m, 2H); 5.85 (s, 1 H); 2.20 (s, 3 H); 2.10 (s, 6H).


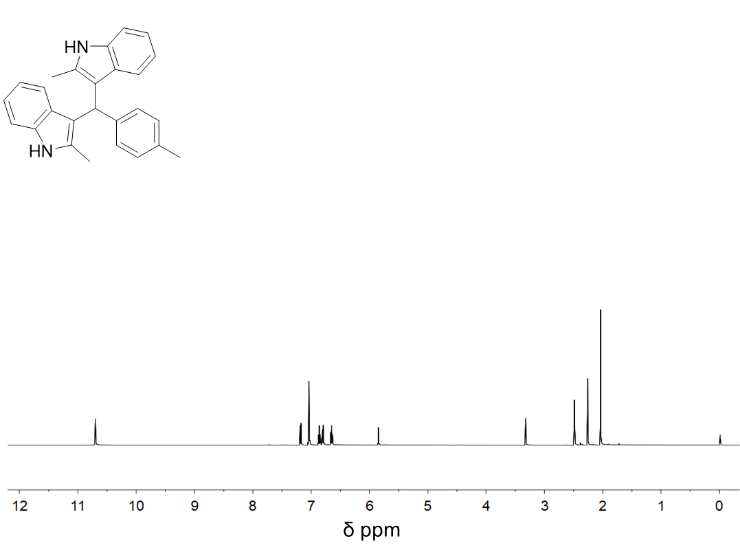


**Phenethyl(*p*-tolyl)sulfane**

^1^H NMR (500 MHz, *d*-chloroform): δ 7.32 – 7.26 (m, 4H), 7.22 (d, J = 9 Hz, 1H), 7.18 (d, J = 9 Hz, 2H), 7.11 (d, J = 10 Hz, 2H), 3.12 (t, J = 9.5 Hz, 2H), 2.90 (t, J = 10.5 Hz, 2H), 2.32 (s, 3H).


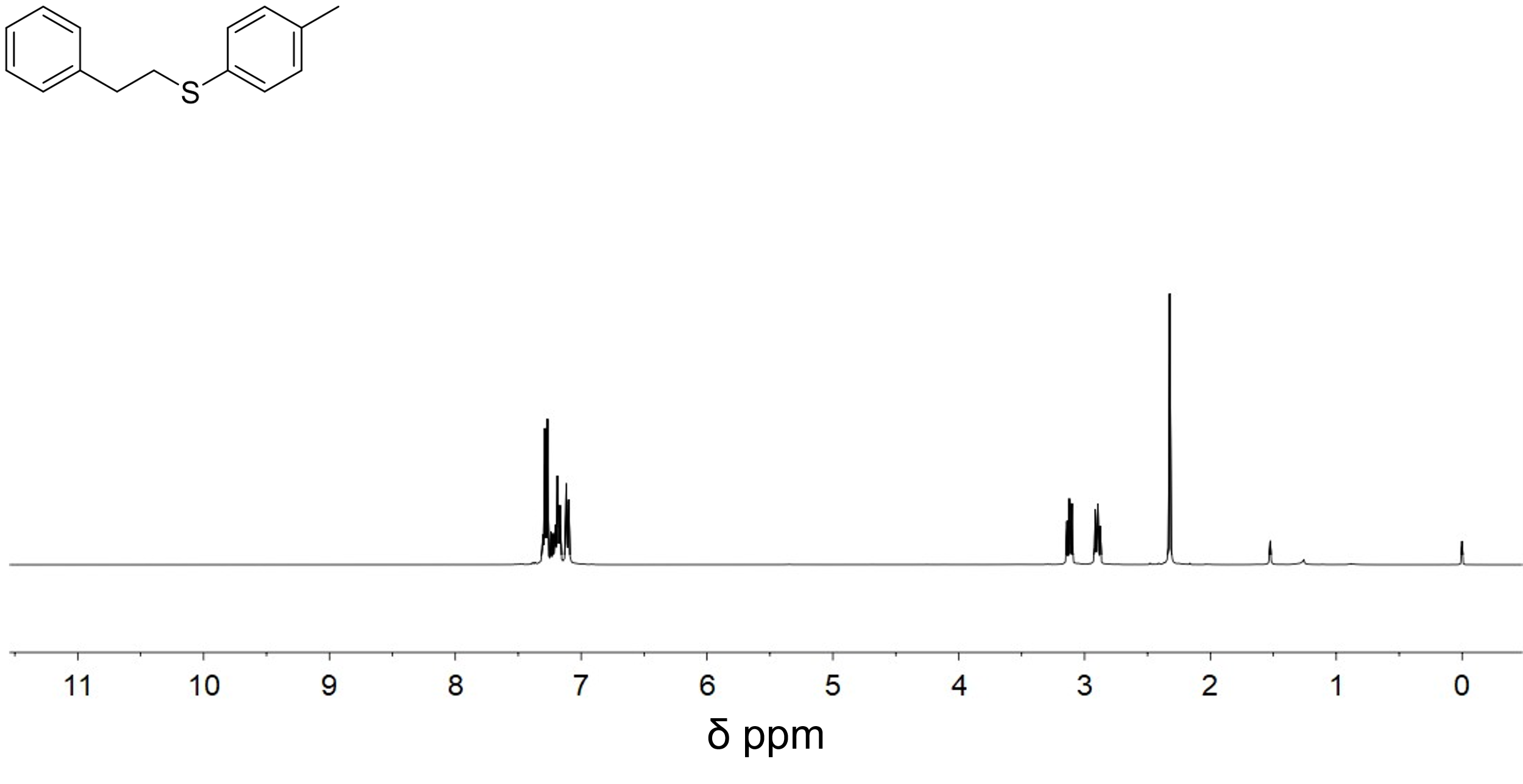


**1-Methyl-4-(phenethylsulfinyl)benzene**

^1^H NMR (500 MHz, *d*-chloroform): δ 7.52 (d, J = 8.2 Hz, 2H), 7.32 (d, J = 8.0 Hz, 2H), 7.27 (d, J = 7.6 Hz, 2H), 7.21 (d, J = 7.3 Hz, 1H), 7.18 – 7.15 (m, 2H), 3.07 – 3.01 (m, 3H), 2.91 – 2.84 (m, 1H), 2.41 (s, 3H).


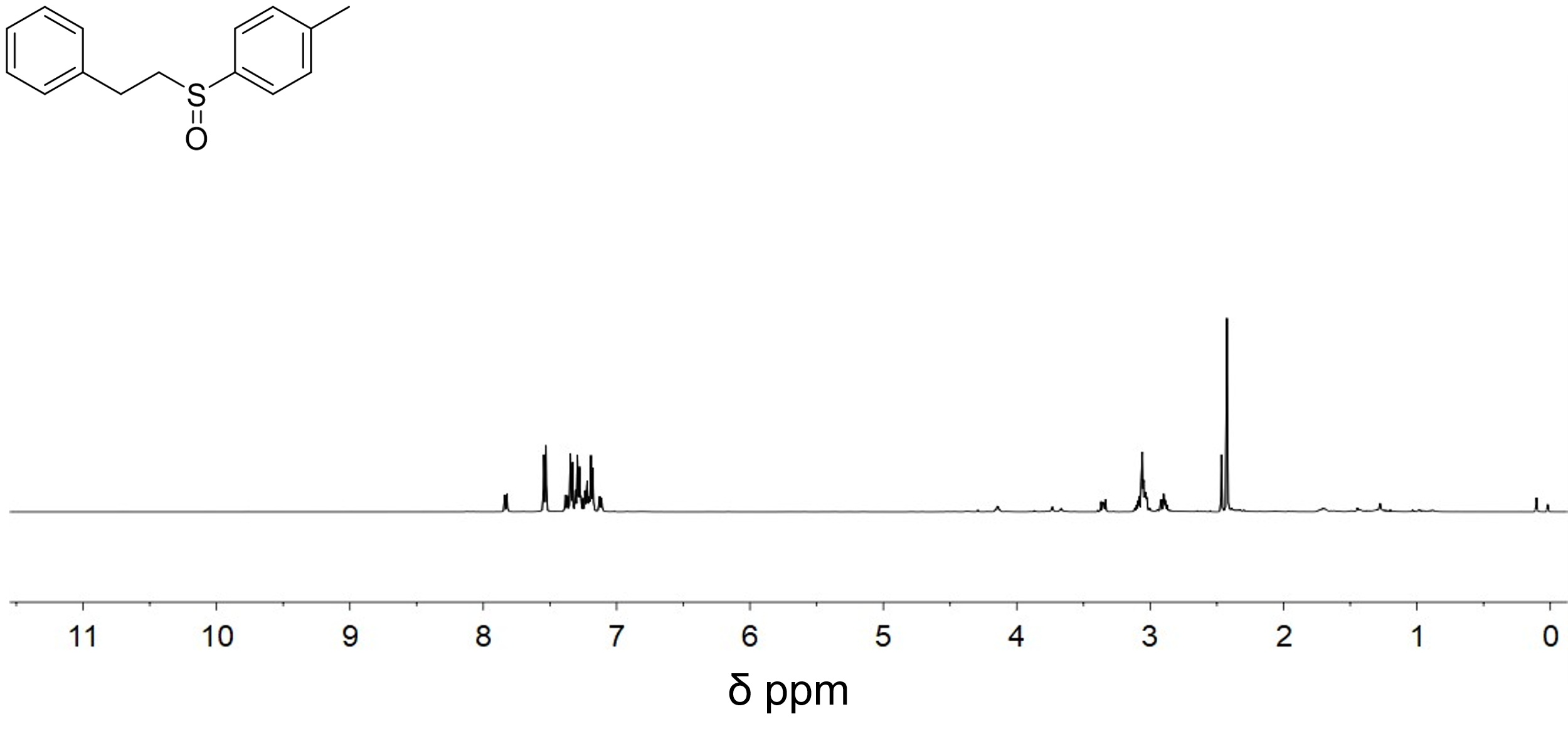


**Supporting Movie 1. Chemical release effect on particle diffusion.** This movie compares particle motion in a glycerol/water film in contact with an LC film under two conditions: (left) without chemical release and (right) with glycerol microdroplet release from the LC film to the glycerol/water film. Both systems are heated to 70°C. The movie highlights the convective flow induced by microdroplet release, contrasting it with the purely Brownian motion observed in the absence of release.

**Supplemental references**

[1] a) J. Schindelin, I. Arganda-Carreras, E. Frise, V. Kaynig, M. Longair, T. Pietzsch, S. Preibisch, C. Rueden, S. Saalfeld, B. Schmid, J.-Y. Tinevez, D. James White, V. Hartenstein, K. Eliceiri, P. Tomancak, A. Cardona, Fiji: An open-source platform for biological-image analysis. *, Nat. Methods* **2012**, *9*, 676–682; b) E. Meijering, O. Dzyubachyk, I. Smal, Methods for cell and particle tracking*, Methods Enzymol.* **2012**, *504*, 183–200.

[2] T. S. Wong, S. H. Kang, S. K. Tang, E. J. Smythe, B. D. Hatton, A. Grinthal, J. Aizenberg, Bioinspired self-repairing slippery surfaces with pressure-stable omniphobicity*, Nature* **2011**, *477*, 443–447.

[3] a) J. D. Smith, R. Dhiman, S. Anand, E. Reza-Garduno, R. E. Cohen, G. H. McKinley, K. K. Varanasi, Droplet mobility on lubricant-impregnated surfaces*, Soft Matter* **2013**, *9*, 1772–1780; b) S. Anand, A. T. Paxson, R. Dhiman, J. D. Smith, K. K. Varanasi, Enhanced condensation on lubricant-impregnated nanotextured surfaces*, ACS Nano* **2012**, *6*, 10122–10129.

[4] M. J. Kreder, D. Daniel, A. Tetreault, Z. Cao, B. Lemaire, J. V. I. Timonen, J. Aizenberg, Film dynamics and lubricant depletion by droplets moving on lubricated surfaces*, Phys. Rev. X* **2018**, *8*, 031053.

[5] E. Q. Li, I. U. Vakarelski, D. Y. Chan, S. T. Thoroddsen, Stabilization of thin liquid films by repulsive van der waals force*, Langmuir* **2014**, *30*, 5162–5169.

[6] a) H. J. Butt, M. Kappl, Surface and interfacial forces*, Wiley-VCH* **2010**; b) F. Schellenberger, J. Xie, N. Encinas, A. Hardy, M. Klapper, P. Papadopoulos, H. J. Butt, D. Vollmer, Direct observation of drops on slippery lubricant-infused surfaces*, Soft Matter* **2015**, *11*, 7617–7626.

[7] a) J. N. Israelachvili, Intermolecular and surface forces*, Elsevier* **2011**; b) C. M. Roth, B. L. Neal, A. M. Lenhoff, van der Waals interactions involving proteins*, Biophys. J.* **1996**, *70*, 977–987.

[8] S. Adera, J. Alvarenga, A. V. Shneidman, C. T. Zhang, A. Davitt, J. Aizenberg, Depletion of lubricant from nanostructured oil-infused surfaces by pendant condensate droplets*, ACS Nano* **2020**, *14*, 8024–8035.

[9] P.-G. de Gennes, F. Brochard-Wyart, D. Quéré, Capillarity and wetting phenomena*, Springer* **2004**.

[10] J. W. Kim, H. Kim, M. Lee, J. J. Magda, Interfacial tension of a nematic liquid crystal/water interface with homeotropic surface alignment*, Langmuir* **2004**, *20*, 8110–8113.

[11] Y. K. Kim, X. Wang, P. Mondkar, E. Bukusoglu, N. L. Abbott, Self-reporting and self-regulating liquid crystals*, Nature* **2018**, *557*, 539–544.

[12] M. C. W. V. Boxtel, M. Wübbenhorst, J. V. Turnhout, C. W. M. Bastiaansen, D. J. Broer, Orientational properties and dynamics of nematic liquid crystals mixed with dendrimers for electro-optical switches*, Liq. Cryst.* **2004**, *31*, 1207–1218.

[13] a) Z. Seidalilir, S. A. Taher, Enhanced electro-chemical characteristics and superior electro-optical switching performance of nematic liquid crystal doped with MgO nanoparticles. *, Opt. Quant Electron.* **2024**, *56*, 1229; b) R. R. Shah, N. L. Abbott, Coupling of the orientations of liquid crystals to electrical double layers formed by the dissociation of surface-immobilized salts*, J. Phys. Chem. B* **2001**, *105*, 4936–4950.

[14] a) O. P. Pishnyak, S. Tang, J. R. Kelly, S. V. Shiyanovskii, O. D. Lavrentovich, Levitation, lift, and bidirectional motion of colloidal particles in an electrically driven nematic liquid crystal*, Phys. Rev. Lett.* **2007**, *99*, 127802; b) S. B. Chernyshuk, B. I. Lev, Theory of elastic interaction of colloidal particles in nematic liquid crystals near one wall and in the nematic cell*, Phys. Rev. E* **2011**, *84*, 011707.

[15] M. Kléman, O. D. Lavrentovich, Soft matter physics: An introduction*, Springer* **2003**.
